# Supplementary material for: Effect of ultrasonography surveillance in patients with liver cancer: a population-based longitudinal study
Source: BMJ Open. 2017 Jun 23;7(6):e015936. doi: 10.1136/bmjopen-2017-015936 (PMC5541579; doi:10.1136/bmjopen-2017-015936)
Supplement: Author's manuscript [file bmjopen-2017-015936.draft_revisions.pdf]

BMJ Open

Effect of Ultrasonography Surveillance on Survival of Patients with Liver Cancer: a Population-Based Longitudinal Study

Journal:	*BMJ Open*
Manuscript ID	bmjopen-2017-015936
Article Type:	Research
Date Submitted by the Author:	12-Jan-2017
Complete List of Authors:	Chiang, Jui-Kun<br>Lin , Chih-Wen<br>Kao, Yee-Hsin; Family Medicine; Tainan Municipal Hospital
**Primary Subject Heading**:	General practice / Family practice
Secondary Subject Heading:	Gastroenterology and hepatology, Epidemiology
Keywords:	PREVENTIVE MEDICINE, Hepatobiliary disease < GASTROENTEROLOGY, Hepatobiliary tumours < ONCOLOGY, PRIMARY CARE

SCHOLARONE™
Manuscripts

For peer review only

Effect of Ultrasonography Surveillance on Survival of Patients with Liver Cancer: a Population-Based Longitudinal Study

Jui-Kun Chiang[1], M.D., MSc, Lin Chih-Wen[2, 3] M.D., MBA, Yee-Hsin Kao[4], M.D.

[1]Department of Family Medicine, Dalin Tzu Chi Hospital, Buddhist Tzu Chi Medical Foundation, Dalin, Chiayi, Taiwan

[2]Department of Medical Imaging, Dalin Tzu Chi Hospital, Buddhist Tzu Chi Medical Foundation, Dalin, Chiayi, Taiwan.

[3]School of Medicine, Tzu Chi University, Hualien, Taiwan.

[4]Department of Family Medicine, Tainan Municipal Hospital, Tainan, Taiwan.

Running head: ultrasonography surveillance on survival for liver cancer patient

Corresponding author: Yee-Hsin Kao: m2200767@gmail.com
Department of Family Medicine
Tainan Municipal Hospital
670 Chung-Te Road
Tainan 701, Taiwan
E-mail: m2200767@gmail.com
Tel: +886 6 260-9926 × 22226
Fax: +886 6 260-9900

E-mail for Jui-Kun Chiang: jkch68@gmail.com
E-mail for Lin Chih-Wen: dalinchiayi@gmail.com

Number of manuscript Pages:
Number of Figures: 4
Number of Tables: 5
Words counts: Abstract: 246, Text: 3037 (introduction: 502, discussion: 847)
Number of References: 41

For peer review only - http://bmjopen.bmj.com/site/about/guidelines.xhtml

Abstract

Objective Liver cancer is a growing global public health problem. Ultrasonography is an imaging tool widely used for the early diagnosis of liver cancer. However, the effect of ultrasonography surveillance (US) on the survival of patients with liver cancer is unknown. This study aimed to examine the association between survival and US frequency during the 2 years preceding liver cancer diagnosis.

Methods This population-based longitudinal study was conducted in Taiwan, a region with high liver cancer incidence, by using the National Health Insurance Research Database. We compared survival between patients receiving US three times or more (M3 group) and those receiving US less than three times (L3 group) during the 2 years preceding liver cancer diagnosis. The significant factors for the M3 group were identified.

Results This study enrolled 4621 patients with liver cancer who died during 1997–2010. The median survival was higher in the M3 group (1.42 years) than in the L3 group (0.51 years). Five-year survival probability was significantly higher in the M3 group (14.4%) than in the L3 group (7.7%). From the multivariate logistic regression model, for the M3 group, the most three significant positive factors were the US indications of viral hepatitis, gallbladder diseases, and kidney urinary bladder diseases, and the significant negative factors were the male sex and the US indication of abdominal pain.

For peer review only - http://bmjopen.bmj.com/site/about/guidelines.xhtml

Conclusion Patients with liver cancer receiving US three times or more during the 2 years preceding liver cancer diagnosis might exhibit a higher 5-year survival probability.

Key words: ultrasonography surveillance (US), liver cancer, survival

Strengths and limitations of this study

A nationwide population-based study was conducted in Taiwan, a high incidence of liver cancer.

We found that the five-year survival probability was significantly higher in those who revived three times or more ultrasonography surveillance (14.4%) than those who received US less than three times (7.7%).

Ultrasonography surveillance three times or more within 2 years may be suggested to general population in the area of high incidence of liver cancer.

No information on cancer stage is available in the dataset, and some crucial potential confounding variables are also not available in the dataset for any statistical adjustment.

For peer review only - http://bmjopen.bmj.com/site/about/guidelines.xhtml

Introduction

Liver cancer is a growing global public health problem and is the fifth and ninth most common cancer in men and women worldwide, respectively, and because of its poor outcome, it is the second most common cause of cancer-related mortality, accounting for nearly 746,000 deaths worldwide in 2012.[1] Eastern and Southeastern Asia, including Taiwan, are regions with a high incidence of liver cancer. In Taiwan, liver cancer has been the second leading cause of cancer-related mortality since 2004 and accounted for 8,116 (18.6%) of 43,665 cancer deaths in 2012.[2] The known risk factors for liver cancer are viral infection (hepatitis B virus [HBV] and hepatitis C virus [HCV] infection), alcohol consumption and Alfa toxins, diabetes, and metabolic (nonalcoholic fatty liver disease and hereditary hemochromatosis) and immune-related (primary biliary cirrhosis and autoimmune hepatitis) diseases.[3] A previous study reported that chronic HBV and HCV infections are the major risk factors for liver cancer.[4] For effective control and treatment of viral hepatitis, two programs have been implemented in Taiwan. The mass vaccination program against HBV has considerably reduced the HBV carrier rate in children and adolescents and consequently reduced the incidence of childhood liver cancer in Taiwan.[5-8] The other program is the pilot program titled “Strengthening of treatment for chronic hepatitis B and C under the National Health Insurance” initiated in October 2003 by the

For peer review only - http://bmjopen.bmj.com/site/about/guidelines.xhtml

Taiwanese Center for Disease Control. The program has made attempts to ensure that patients with chronic hepatitis B and C are registered, so that they can benefit from appropriate diagnosis, monitoring, and treatment.[9] Accordingly, the incidence of liver cancer significantly decreased by an average annual percentage change of 1.1% from 2002 to 2012, and the 5-year relative survival rates for liver cancer were 52.0% for stage I, 2.9% for stage IV, and 28.9% for all stages.[10]

The diagnosis of hepatocellular cancer is based on a combination of radiologic, serologic, and histopathologic criteria. Studies have reported that alpha-fetoprotein determination lacks adequate sensitivity and specificity for effective surveillance.[11 12] Thus, surveillance must be based on ultrasonography, and the recommended screening interval is 6 months.[13 14] A previous study reported that for the detecting early-stage liver cancer, ultrasonography had a sensitivity of approximately 63% and a high specificity of approximately 85–90%.[12] Moreover, ultrasonography is a noninvasive and safe method, is well accepted by patients, and is relatively inexpensive.

Previous studies have reported that most patients with hepatocellular carcinoma exhibit low survival. However, patients with early-stage hepatocellular carcinoma who receive potentially curative therapy exhibit considerably improved survival (5-year survival = 40%–70%).[15] A systemic review provided very-low-strength evidence about the effects of hepatocellular carcinoma screening on mortality in

For peer review only - http://bmjopen.bmj.com/site/about/guidelines.xhtml

patients with chronic hepatitis.[16] Regular surveillance with abdominal ultrasonography might facilitate the early detection of liver cancer, thus improving the survival of at-risk patients. This study examined the effect of ultrasonography surveillance (US) on survival and the association between survival and the frequency of ultrasonography during the 2 years preceding liver cancer diagnosis and identified the predictors of receiving more US preceding liver cancer diagnosis.

Methods

Data source and patient identification

In this nationwide population-based study, we analyzed the data from the National Health Insurance Research Database (NHIRD) of Taiwan. The NHI program, established in 1995, is a single-payer health insurance system that covered up to 99.9% of all residents of Taiwan in 2012. Moreover, 97% of medical providers nationwide are affiliated with the program.[17] The Longitudinal Health Insurance Database (LHID) is a nationwide representative database containing all original claims data for the period of 1996–2011 for one million NHI beneficiaries randomly sampled from the 23.22 million NHI enrollees. The availability of such a population-based database has stimulated and facilitated academic research in various scientific disciplines, particularly in the field of health research.[18] The accuracy of

For peer review only - http://bmjopen.bmj.com/site/about/guidelines.xhtml

diagnoses registered in the NHIRD has been validated using five-digit International Classification of Diseases, Ninth Revision, Clinical Modification (ICD-9-CM) codes, including those for diabetes and cancer, indicating the reliability of the data source.[19] In Taiwan, all cancer patients are designated as having a catastrophic illness. We identified liver cancer patients with catastrophic illness designations in LHID2000 and followed up these patients until December 2011. The ICD-9-CM and A codes were used to define liver cancer (155, 155.0, 155.1, A095).

Study sample

This study included patients aged ≥18 years with a first-time diagnosis of liver cancer between January 1, 1997, and December 31, 2010. The NHIRD was used to identify patients with liver cancer, and the cancer cases were confirmed using the Registry of Catastrophic Illness. Patients were excluded if they made no insurance claims during their final year of life, were still alive during half a year before the end of the dataset, or had missing data. A total of 4621 patients with liver cancer were enrolled in this study (Figure 1).

Definition of frequency of abdominal ultrasonography surveillance during 2 years preceding liver cancer diagnosis

We classified the study population into two groups based on to the frequency of

For peer review only - http://bmjopen.bmj.com/site/about/guidelines.xhtml

US during the 2 years preceding liver cancer diagnosis: L3 group (patients with liver cancer who received US less than three times; n = 3149) and M3 group (those who revived regular US, i.e., three times or more; n = 1472).

We classified the diseases that are the indications for ultrasonography into seven different groups by using the ICD-9-CM diagnosis codes. The seven disease groups were as follows: (1) liver-related diseases including chronic hepatitis, cirrhosis, and other liver disorders (ICD-9 codes: 571, 571.x, 572, 5720, 573, and 573.x); (2) viral hepatitis infection (ICD-9 codes for HBV: V0261, 070.20–070.23, and 070.30–070.33; for HCV: V0262, 070.41, 070.44, 070.51, and 070.54); (3) gallbladder and biliary tract-related diseases (ICD-9 codes: 574–576); (4) abdominal pain conditions (ICD-9 codes: 789.0x, 789.4x, 789.6x, and 789.9); (5) kidney–urinary–bladder (KUB) diseases (ICD-9 codes: 591, 592, 592.0, 592.1, 592.9, 593, and 593.2); (6) pancreatic diseases (ICD-9 code: 577.x); and (7) other conditions such as hepatomegaly, splenomegaly, abdominal or pelvic mass, and ascites (ICD-9 codes: 7891, 7892, 7893x,and 7895).

Definition of variables

Charlson comorbidity index (CCI) was calculated by examining ICD-9-CM diagnosis and procedure codes recorded in the year before diagnosis, according to the Deyo method, and CCI was applied to inpatient and outpatient claims, according to

For peer review only - http://bmjopen.bmj.com/site/about/guidelines.xhtml

the method of Klabundle *et al.*[20-22]

The study protocol was reviewed and approved by the Research Ethics Committee of Dalin Tzu Chi Hospital, Buddhist Tzu Chi Medical Foundation, Taiwan (Approval No. B10304018). Because the NHIRD contains only deidentified secondary data, the review board waived the requirement for informed consent.

Statistical analyses

Continuous variables are expressed in mean ± standard deviation (SD), and categorical variables are presented in frequency and percentage. In univariate analysis, two-sample *t* test, Wilcoxon rank-sum test, chi-square test, and Fisher's exact test were used to examine the differences in the distributions of continuous variables and categorical variables between the two groups. The survival duration (years) was defined as the duration from the day of diagnosis to the day of death. Generalized additive models (GAMs) were fitted to detect the potential nonlinear effects of continuous covariates and to identify the appropriate cutoff points for discretizing continuous covariates, if necessary, during the stepwise variable selection. Computationally, the VGAM function (with the default values of smoothing parameters) of the VGAM package was used to fit GAMs for the binary outcome in R.

Cox proportional hazards regression models were used to estimate the

For peer review only - http://bmjopen.bmj.com/site/about/guidelines.xhtml

event-specific hazard ratios (HRs) and 95% confidence intervals (CIs) of all the variables listed in Tables 1 and 2. Subsequently, multivariate analysis was conducted by fitting multiple logistic regression models with the stepwise variable selection procedure to identify the crucial predictors of receiving US three times or more during the 2 years preceding liver cancer diagnosis. The goodness-of-fit (GOF) of the final logistic regression model was assessed by estimating area under the receiver operating characteristic (ROC) curve (AUC; also called the c statistic), where $0 \leq c \leq 1$, and the Hosmer–Lemeshow GOF test. In practice, $c \geq 0.7$ suggests an acceptable level of discrimination power for a fitted logistic regression model. Moreover, in the Hosmer–Lemeshow test, $p > 0.05$ indicates a good fit for the logistic regression model. The variance inflating factor of ≥10 for continuous covariates or ≥2.5 for categorical covariates indicate the occurrence the multicollinearity problem among some covariates in the fitted logistic regression model. Statistical analysis was performed using the R 3.0.2 software (R Foundation for Statistical Computing, Vienna, Austria). Two-sided $p \leq 0.05$ was considered significant.

Results

We enrolled 4621 adult patients (3287 men and 1334 women; ratio = 2.46:1) with liver cancer who died during 1997–2010. Figure 1 depicts the study design. We

For peer review only - http://bmjopen.bmj.com/site/about/guidelines.xhtml

explored the association between the frequency of ultrasonography during the 2 years preceding liver cancer diagnosis and 5-year survival probability. The optimal cutoff point for receiving US during the 2 years preceding liver cancer diagnosis was set at three times for higher 5-year survival probability (Figure 2).

Table 1 summarizes of the demographic characteristics of the study patients and indicates that the patients in the M3 group were more likely to be female (p < 0.001) and were more likely to have the comorbidities of viral hepatitis (HBV or HCV infection) (p < 0.001), cirrhosis (p < 0.001), diabetes (p < 0.001), hypertension (p < 0.001), and stroke (p = 0.001). The median survival of the M3 group (1.42 years) was higher than that of the L3 group (0.51 years) (p < 0.001). Table 2 summarizes the indications for abdominal US used in this study. The most frequent indications were viral hepatitis (2580, 55.8%), gallbladder diseases (1690, 36.6%), and abdominal pain (1387, 30.0%). In this study, all indications, except for abdominal pain (p = 0.098), were significantly higher in the M3 group than in the L3 group. Compared with the L3 group, significantly higher proportions of the M3 group received all treatments for liver cancer, except for radiotherapy (p = 0.097), and the M3 group had significantly higher 5-year survival probability (7.7% and 14.4%, respectively; p < 0.001) (Table 3). From the multivariate Cox proportional hazards regression model, the positive significant predictors of the death event were the male sex (HR: 1.14, 95% CI:

For peer review only - http://bmjopen.bmj.com/site/about/guidelines.xhtml

1.07–1.22), age (per 5 years) (HR: 1.02, 95% CI: 1.01–1.03), diabetes (HR: 1.13, 95% CI: 1.04–1.22), and high CCI score (per the score of 1) (HR: 1.07, 95% CI: 1.05–1.08). Moreover, the negative significant predictors of the death event were hypertension (HR: 0.83, 95% CI: 0.78–0.89), stroke (HR: 0.78, 95% CI: 0.72–0.84), chronic kidney diseases (CKDs) (HR: 0.84, 95% CI: 0.74–0.92), viral hepatitis (HBV or HCV infection) (HR: 0.79, 95% CI: 0.72–0.88), receiving US three times or more during the 2 years preceding liver cancer diagnosis (HR: 0.80, 95% CI: 0.75-0.85). The significant indications for US included KUB diseases (HR: 0.92, 95% CI: 0.86–0.995), gallbladder diseases (HR: 0.90, 95% CI: 0.85–0.96), chronic hepatitis (except for HBV or HCV infection) (HR: 0.90, 95% CI: 0.81–0.999), and pancreatic diseases (HR: 0.80, 95% CI: 0.71–0.91). Moreover, the significant complications of liver cancer were upper gastrointestinal bleeding (gastric ulcer or duodenal ulcer) (HR: 0.92, 95% CI: 0.86–0.98) and esophageal variceal bleeding (HR: 0.90, 95% CI: 0.83–0.97), and the significant treatments received for liver cancer were transcatheter arterial chemoembolization (TACE) (HR: 0.82, 95% CI: 0.76–0.89), radiotherapy (HR: 0.81, 95% CI: 0.75–0.87), radiofrequency ablation (RFA) (HR: 0.79, 95% CI: 0.68–0.93), chemotherapy (HR: 0.71, 95% CI: 0.67–0.76), resection (HR: 0.67, 95% CI: 0.61–0.73), and percutaneous ethanol injection (PEI) (HR: 0.64, 95% CI: 0.57–0.70). The significant demographic factors were employment (HR: 0.89, 95% CI:

For peer review only - http://bmjopen.bmj.com/site/about/guidelines.xhtml

0.83-0.95) and living in Northern Taiwan (HR: 0.91, 95% CI: 0.85-0.97) (Table 4). Survival probability was significantly higher in the M3 group than in the L3 group (*p* < 0.001; Figure 3). From the multivariate logistic regression model, the significant positive factors for the M3 group were viral hepatitis (HBV or HCV infection) (odds ratio [OR]: 3.66, 95% CI: 2.76–4.85), gallbladder diseases (OR: 1.82, 95% CI: 1.59–2.09), KUB diseases (OR: 1.80, 95% CI: 1.53–2.11), pancreatic diseases (OR: 1.79, 95% CI: 1.37–2.34), chronic hepatitis (except for viral hepatitis; OR: 1.75, 95% CI: 1.31–2.34), CKD (OR: 1.60, 95% CI: 1.30–1.96), diabetes (OR: 1.56, 95% CI: 1.31–1.86), the US indication of other conditions (OR: 1.47, 95% CI: 1.29-1.69), hypertension (OR: 1.17, 95% CI: 1.01–1.34), living in Central Taiwan (OR: 1.16, 95% CI: 1.01–1.34). Moreover, the significant negative factors were the male sex (OR: 0.60, 95% CI: 0.52–0.69) and the US indication of abdominal pain (OR: 0.81, 95% CI: 0.70–0.93) (Table 5). In the Hosmer–Lemeshow test, the *p* value of 0.156 indicated a good fit, and the AUC was acceptable (0.707, 95% CI: 0691–0723) (Figure 4).

Discussion

In this study, we found that patients with liver cancer who received US three times or more during the 2 years preceding liver cancer diagnosis exhibited high 5-year survival, (14.4% vs. 7.7%). We also found that compared with the L3 group, higher proportions of the M3 group received treatment for early stage of liver cancer,

For peer review only - http://bmjopen.bmj.com/site/about/guidelines.xhtml

such as PEI, hepatectomy, RFA, and liver transplantation. The generalisability of ultrasonography surveillance three times or more within 2 years may be suggested to general population in the area of high incidence of liver cancer.

One previous trial also reported a similar result, in which biannual US for patients with hepatitis B virus infection reduced hepatocellular carcinoma mortality by 37%.[23] Another cohort study reported that patients with viral hepatitis who received routine US exhibited higher 5-year survival than those who did not (31.84% and 20.67%, respectively).[24] Moreover, a trial reported that early-stage hepatocellular carcinoma was more likely to be detected in patients with viral hepatitis receiving US at a 4-month interval than in those receiving US at a 12-month interval; however, the overall survival was not different at the 4-year follow-up.[25] A systemic review reported that the effects of hepatocellular carcinoma screening on mortality in patients with chronic hepatitis were uncertain.[16] Thus, receiving US at a high frequency had no beneficial effect on the 5-year survival probability for patients with hepatocellular carcinoma. This finding may be because the early detection of liver cancer improves the rate of curative treatments. However, the further, regular follow-up for possible recurrences and treatments is vital. The prognosis of patients with liver cancer is not solely related to the tumor stage.[26] Although the survival of patients with liver cancer has improved in the past decade, physicians caring for at-risk patients need to provide

For peer review only - http://bmjopen.bmj.com/site/about/guidelines.xhtml

regular high-quality screening, including ultrasonography.[13]

Although advanced diagnostic techniques such as dynamic multiphasic multidetector-row computed tomography and magnetic resonance imaging are the standard diagnostic methods for the noninvasive diagnosis of liver cancer, ultrasonography still plays a crucial role in liver cancer surveillance.[27] The recommended interval for US screening is 6 months for patients with viral hepatitis infection and 3 months for patients with cirrhosis in Taiwan. Ultrasonography can be performed in hospital and clinics, if indicated, and the cost of each ultrasonography screening (USD $23.1) is covered by the NHI program in Taiwan.

In this study, we found that patients with chronic hepatitis (HBV, HCV infection, and non-B or non-C hepatitis), diabetes, CKD, cerebral vascular accident (CVA), hypertension, gallbladder diseases, KUB diseases, pancreatic diseases, and other conditions (i.e., hepatomegaly, splenomegaly, abdominal or pelvic mass, and ascites) were more likely to receive US three times or more during the 2 years preceding liver cancer diagnosis. One reason that patients with diabetes were more likely to receive US three times or more in this study might be that these patients are at a high risk of primary liver cancer,[28-30] and the association between diabetes and liver cancer is significantly strengthened when the history of diabetes is longer than 10 years.[31 32] One reason for those patients with CKD were more likely to receive US three times or

For peer review only - http://bmjopen.bmj.com/site/about/guidelines.xhtml

more in this study might be the close link between the kidney and cancer,[33] and cancer has become increasingly recognized as a complication and a major cause of morbidity and mortality in the CKD population.[34] The reason that patients with comorbidities such as CVA, hypertension, gallbladder diseases, KUB diseases, pancreatic diseases, and other conditions were more likely to receive US three times or more in this study might be that patients with more comorbidities and physician visits probably undergo more laboratory tests and imaging studies, including US, if they experience any discomfort.

In this study, compared with the L3 group, higher proportions of the M3 group received treatments for early-stage liver cancer, such as PEI, hepatectomy, RFA, and liver transplantation. These treatments were reported as therapies for early-stage hepatocellular carcinoma in previous studies.[35-38] We also found that compared with the L3 group, higher proportions of the M3 group received TACE, although the therapeutic effects of TACE for early-stage hepatocellular carcinoma are unclear .[39]

A previous study reported that mass screening for liver cancer by using US in high-endemic regions, including Taiwan, is cost-effective and recommended.[40] In this study, the significant positive factors for the M3 group were viral hepatitis, gallbladder diseases, KUB diseases, pancreatic diseases, chronic hepatitis (except for viral infection), diabetes, other indications for US (hepatomegaly, splenomegaly, etc.),

For peer review only - http://bmjopen.bmj.com/site/about/guidelines.xhtml

hypertension, and living in Central Taiwan. However, the significant negative factors for the M3 group were the male sex and the US indication of abdominal pain. The reason might be that men have fewer intentions for medical service than women.[41] The US indication of abdominal pain might be a significant negative factor because after seeking medical service for one event and after solving the problem, patients do not return for subsequent follow-up. However, the precise reason for this indication is not available from the claims data, and this is a limitation of this study. Other limitations of this study are that no information on cancer stage is available in the dataset, and some crucial potential confounding variables, such as body mass index, smoking, and alcohol intake, are also not available in the dataset for any statistical adjustment.

Conclusions

Five-year survival probability might improve in patients with liver cancer who receive abdominal US three times or more during the 2 years preceding liver cancer diagnosis. Additional studies should prospectively investigate whether abdominal US can be used to identify early-stage liver cancer and then examine whether further treatment with regular follow-up improves survival.

Acknowledgements

For peer review only - http://bmjopen.bmj.com/site/about/guidelines.xhtml

This study is based in part on data from the National Health Insurance Research Database provided by the Bureau of National Health Insurance, Department of Health and managed by the National Health Research Institutes. The interpretation and conclusions contained herein do not represent those of the Bureau of National Health Insurance, Department of Health or National Health Research Institutes.

Footnotes

Contributors: JKC, LCW and YHK designed, conducted and drafted the manuscript. JKC analyzed the data. All authors contributed to the manuscript, revised drafts critically for important intellectual content, and read and approved the final manuscript.

Funding: JK Chiang received research grants from Dalin Tzu Chi Hospital, Buddhist Tzu Chi Medical Foundation (DTCRD105 (2)-E-22). The funding source had no involvement in study design; in the collection, analysis and interpretation of data; in the writing of the report; and in the decision to submit the article for publication.

Competing interests: None declared.

Ethics approval: The study protocol was reviewed and approved by the Research Ethics Committee of Dalin Tzu Chi Hospital, Buddhist Tzu Chi Medical Foundation, Taiwan (Approval No. B10304018).

Data sharing statement: No additional data are available.

For peer review only - http://bmjopen.bmj.com/site/about/guidelines.xhtml

Table 1. Comparison of demographic characteristics between the L3 group and the M3 group

Variable	L3 group, n (%)	M3 group, n (%)	*p* value
Total number (%)	3149 (68.1)	1472 (31.9)	
Age (years)	63.04 ± 13.92	63.77 ± 11.76	0.474
Gender			< 0.001
female	807 (25.6)	527 (35.8)	
male	2342 (74.4)	945 (64.2)	
Survival*	0.51 (0.17, 1.81)	1.42 (0.48, 3.34)	< 0.001
HBV	1119 (35.5)	619 (42.1)	< 0.001
HCV	660 (21.0)	620 (42.1)	< 0.001
Diabetes	535 (17.0)	361 (24.5)	< 0.001
CKD	276 (8.8)	232 (15.8)	< 0.001
CVA	535 (17.0)	297 (20.2)	0.001
Hypertension	1173 (37.2)	691 (46.9)	< 0.001
Cirrhosis	2048 (65.0)	1183 (80.4)	< 0.001
Employment (yes)	1873 (59.5)	891 (60.5)	0.499
Northern area in Taiwan	1063 (33.8)	467 (31.7)	0.178
Central area in Taiwan	888 (28.2)	461 (31.3)	0.031
Southern area in Taiwan	1032 (32.8)	480 (32.6)	0.920
Eastern area in Taiwan	153 (4.9)	57 (3.9)	0.150

Abbreviations: HBV, hepatitis B virus; HCV, hepatitis C virus; CKD, chronic kidney disease; CVA, cerebral vascular accident; CCI: Charlson comorbidity index

Survival*: median (first quartile, third quartile)

For peer review only

For peer review only - http://bmjopen.bmj.com/site/about/guidelines.xhtml

Table 2. Ultrasonography surveillance indications in the L3 group and the M3 group

Variable	Total number (%)	L3 group, n (%)	M3 group, n (%)	*p* value
Viral hepatitis (HBV, HCV)	2580 (55.8)	1556 (49.4)	1024 (69.6)	< 0.001
Chronic hepatitis (except HBV, HCV)	1560 (33.8)	1179 (37.4)	381 (25.9)	< 0.001
Gall bladder diseases	1690 (36.6)	989 (31.4)	701 (47.6)	< 0.001
Abdominal pain	1387 (30.0)	921 (29.2)	466 (31.7)	0.098
KUB diseases	928 (20.1)	524 (16.6)	404 (27.4)	< 0.001
Pancreas diseases	271 (5.9)	133 (4.2)	138 (9.4)	< 0.001
Others*	1780 (38.5)	1085 (34.5)	695 (47.2)	< 0.001

Abbreviations: HBV, hepatitis B virus; HCV, hepatitis C virus; CKD, chronic kidney disease; CVA, cerebral vascular accident; CCI, Charlson comorbidity index; DU, duodenal ulcer; GU, gastric ulcer; EV; esophageal varices; US, ultrasonography surveillances

Others* include hepatomegaly, splenomegaly, abdominal or pelvic mass, and ascites.

For peer review only - http://bmjopen.bmj.com/site/about/guidelines.xhtml

Table 3. Comparison of treatments for ultrasonography surveillance between the L3 group and the M3 group

Variable	L3 group, n (%)	M3 group, n (%)	*p* value
PEI	200 (6.4)	244 (16.6)	< 0.001
Hepatectomy	343 (10.9)	221 (15.0)	< 0.001
RFA	71 (2.3)	101 (6.9)	< 0.001
Liver transplantation	2 (0.1)	6 (0.4)	0.015
TACE	584 (18.5)	453 (30.8)	< 0.001
Chemotherapy	1169 (37.1)	677 (46.0)	< 0.001
Radiotherapy	647 (20.5)	334 (22.7)	0.097
5 years survival probability	7.7%	14.4%	< 0.001

Abbreviations: HBV, hepatitis B virus; HCV, hepatitis C virus; CKD, chronic kidney disease; CVA, cerebral vascular accident; CCI, Charlson comorbidity index; DU, duodenal ulcer; GU, gastric ulcer; EV; esophageal varices; PEI, percutaneous ethanol injection; RFA, radiofrequency ablation; TACE, transcatheter arterial chemoembolization; UGI, upper gastrointestinal tract.

For peer review only

Table 4. Multivariate Cox proportional hazards regression model of significant factors of the death event among patients with liver cancer

Variable	H.R.	95% C.I.	*p* value
Male	1.14	1.07-1.22	<0.001
Age (HCC diagnosis) per 5 years	1.02	1.01-1.03	0.002
Diabetes	1.13	1.04-1.22	0.004
Hypertension	0.83	0.78-0.89	<0.001
CVA	0.78	0.72-0.84	<0.001
CKD	0.84	0.74-0.92	<0.001
Viral hepatitis (HBV, HCV)	0.79	0.72-0.88	<0.001
CCI (per 1 score)	1.07	1.05-1.08	<0.001
US indications			
3 times or more US	0.80	0.75-0.85	<0.001
KUB diseases	0.92	0.86-0.995	0.037
Gall bladder diseases	0.90	0.85-0.96	0.001
Chronic liver diseases (except HBV, HCV)	0.90	0.81-0.999	0.047
Pancreas diseases	0.80	0.71-0.91	<0.001
Complication of liver cancer			
UGI bleeding (GU, DU)	0.92	0.86-0.98	0.009
EV bleeding	0.90	0.83-0.97	0.008
Treatments			
TACE	0.82	0.76-0.89	<0.001
Radiotherapy	0.81	0.75-0.87	<0.001
RFA	0.79	0.68-0.93	0.004
Chemotherapy	0.71	0.67-0.76	<0.001
hepatectomy	0.67	0.61-0.73	<0.001
PEI	0.64	0.57-0.70	<0.001
Background			
Employment	0.89	0.83-0.95	<0.001
Northern area in Taiwan	0.91	0.85-0.97	0.003

Abbreviations: HBV, hepatitis B virus; HCV, hepatitis C virus; CKD, chronic kidney disease; CVA, cerebral vascular accident; CCI, Charlson comorbidity index; DU, duodenal ulcer; GU, gastric ulcer; EV; esophageal varices; PEI, percutaneous ethanol injection; RFA, radiofrequency ablation; TACE, transcatheter arterial chemoembolization; UGI, upper gastrointestinal tract; US, ultrasonography surveillances

For peer review only - http://bmjopen.bmj.com/site/about/guidelines.xhtml

Table 5. Multivariate logistic regression model of the factors associated with receiving ultrasonography surveillance three times or more (M3 group) during 2 years preceding liver cancer diagnosis

Variables	O.R.	95% C.I.	p value
Male	0.60	0.52-0.69	<0.001
Diabetes	1.56	1.31-1.86	<0.001
Hypertension	1.17	1.01-1.34	0.03
CKD	1.60	1.30-1.96	<0.001
US indications			
Viral hepatitis (HBV or HCV infection)	3.66	2.76-4.85	<0.001
Gall bladder diseases	1.82	1.59-2.09	<0.001
KUB diseases	1.80	1.53-2.11	<0.001
Pancreas diseases	1.79	1.37-2.34	<0.001
Chronic hepatitis except viral hepatitis	1.75	1.31-2.34	<0.001
Other*	1.47	1.29-1.69	<0.001
Abdominal pain	0.81	0.70-0.93	0.004
Central area in Taiwan	1.16	1.01-1.34	0.04

Abbreviations: HBV, hepatitis B virus; HCV, hepatitis C virus; CKD, chronic kidney disease; CVA, cerebral vascular accident; CCI: Charlson comorbidity index; KUB: kidney urinary bladder; US, ultrasonography surveillance

US indication: other* includes hepatomegaly, splenomegaly, abdominal or pelvic mass, and ascites.

For peer review only

Figure Legends

Figure 1. The study flowchart.

Figure 2. Smoothing curve of the frequency of ultrasonography during 2 years preceding liver cancer against 5-year survival probability after adjustment. (*x*-axis: frequency of ultrasonography during 2 years preceding liver cancer; *y*-axis: p, probability of 5-year survival.)

Figure 3. Survival curves stratified by two groups listed in Table 1 (less than 3 times [L3 group] and 3 times or more [M3 group] ultrasonography surveillances during 2 years preceding liver cancer diagnosis)

Figure 4. Receiver operating characteristic curves for significant factors listed in Table 5 for predicting receiving ultrasonography three or more times during 2 years preceding liver cancer diagnosis

For peer review only - http://bmjopen.bmj.com/site/about/guidelines.xhtml

References

1. GLOBOCAN 2012: Estimated Cancer Incidence, Mortality and Prevalence Worldwide in 2012. Available at : http://globocan.iarc.fr/Pages/fact_sheets_cancer.aspx. Accessed July 6, 2016.

2. Ministry of Health and Welfare. Statistics of Causes of Death, Volume1. Available at : http://www.mohw.gov.tw/cht/DOS/Statistic.aspx?f_list_no=312&fod_list_no=2747 Accessed January 18, 2016.

3. Parikh S, Hyman D. Hepatocellular cancer: a guide for the internist. *Am J Med* 2007; 120: 194-202.

4. Gomaa AI, Khan SA, Toledano MB, et al. Hepatocellular carcinoma: epidemiology, risk factors and pathogenesis. *World J Gastroenterol* 2008; 14: 4300-4308.

5. Chen DS, Hsu NH, Sung JL, et al. A mass vaccination program in Taiwan against hepatitis B virus infection in infants of hepatitis B surface antigen-carrier mothers. *JAMA* 1987; 257: 2597-2603.

6. Liu CJ, Kao JH. Hepatitis B virus-related hepatocellular carcinoma: epidemiology and pathogenic role of viral factors. *J Chin Med Assoc* 2007; 70: 141-145.

7. Ni YH, Chang MH, Huang LM, et al. Hepatitis B virus infection in children and adolescents in a hyperendemic area: 15 years after mass hepatitis B vaccination. *Ann Intern Med* 2001; 135: 796-800.

8. Chang MH, Chen CJ, Lai MS, et al. Universal hepatitis B vaccination in Taiwan and the incidence of hepatocellular carcinoma in children. Taiwan Childhood Hepatoma Study Group. *N Engl J Med* 1997; 336: 1855-1859.

9. Wait S, Chen DS. Towards the eradication of hepatitis B in Taiwan. *Kaohsiung J Med Sci* 2012; 28: 1-9.

10. Chiang C-J, Lo W-C, Yang Y-W, et al. Incidence and survival of adult cancer patients in Taiwan, 2002-2012. J Formos Med Assoc. (2015), http://dx.doi.org/10.1016/j.jfma.2015.10.011

11. Lok AS, Sterling RK, Everhart JE, et al. Des-gamma-carboxy prothrombin and alpha-fetoprotein as biomarkers for the early detection of hepatocellular carcinoma. *Gastroenterology* 2010; 138: 493-502.

12. Singal A, Volk ML, Waljee A, et al. Meta-analysis: surveillance with ultrasound for early-stage hepatocellular carcinoma in patients with cirrhosis. *Aliment Pharmacol Ther* 2009; 30: 37-47.

13. Bruix J, Sherman M, American Association for the Study of Liver D.

For peer review only

Management of hepatocellular carcinoma: an update. *Hepatology* 2011; 53: 1020-1022.

14. EASL-EORTC clinical practice guidelines: management of hepatocellular carcinoma. *J Hepatol* 2012; 56: 908-943.

15. El-Serag HB, Davila JA. Surveillance for hepatocellular carcinoma: in whom and how? *Therap Adv Gastroenterol* 2011; 4: 5-10.

16. Kansagara D, Papak J, Pasha AS, et al. Screening for hepatocellular carcinoma in chronic liver disease: a systematic review. *Ann Intern Med* 2014; 161: 261-269.

17. National Health Insurance Research Database (NHIRD), Taiwan. http://nhird.nhri.org.tw/date_01.html Accessed May 22, 2016.

18. Chen YC YH, Wu JC, Haschler I, Chen TJ, Wetter T. Taiwan's National Health Insurance Research Database: administrative health care database as study object in bibliometrics. *Scientometrics* 2011; 86(2): 365-380.

19. Lin CC, Lai MS, Syu CY, et al. Accuracy of diabetes diagnosis in health insurance claims data in Taiwan. *J Formos Med Assoc* 2005; 104: 157-163.

20. Charlson ME, Pompei P, Ales KL, et al. A new method of classifying prognostic comorbidity in longitudinal studies: development and validation. *J Chronic Dis* 1987; 40: 373-383.

21. Deyo RA, Cherkin DC, Ciol MA. Adapting a clinical comorbidity index for use with ICD-9-CM administrative databases. *J Clin Epidemiol* 1992; 45: 613-619.

22. Klabunde CN, Potosky AL, Legler JM, et al. Development of a comorbidity index using physician claims data. *J Clin Epidemiol* 2000; 53: 1258-1267.

23. Zhang BH, Yang BH, Tang ZY. Randomized controlled trial of screening for hepatocellular carcinoma. *J Cancer Res Clin Oncol* 2004; 130: 417-422.

24. Thein HH, Campitelli MA, Yeung LT, et al. Improved Survival in Patients with Viral Hepatitis-Induced Hepatocellular Carcinoma Undergoing Recommended Abdominal Ultrasound Surveillance in Ontario: A Population-Based Retrospective Cohort Study. *PLoS One* 2015; 10: e0138907.

25. Wang JH, Chang KC, Kee KM, et al. Hepatocellular carcinoma surveillance at 4- vs. 12-month intervals for patients with chronic viral hepatitis: a randomized study in community. *Am J Gastroenterol* 2013; 108: 416-424.

26. Greten TF, Papendorf F, Bleck JS, et al. Survival rate in patients with hepatocellular carcinoma: a retrospective analysis of 389 patients. *Br J Cancer* 2005; 92: 1862-1868.

27. Lee JM, Yoon JH, Kim KW. Diagnosis of hepatocellular carcinoma: newer radiological tools. *Semin Oncol* 2012; 39: 399-409.

28. Adami HO, Chow WH, Nyren O, et al. Excess risk of primary liver cancer in patients with diabetes mellitus. *J Natl Cancer Inst* 1996; 88: 1472-1477.

For peer review only

For peer review only - http://bmjopen.bmj.com/site/about/guidelines.xhtml

29. La Vecchia C, Negri E, Decarli A, et al. Diabetes mellitus and the risk of primary liver cancer. *Int J Cancer* 1997; 73: 204-207.

30. Fujino Y, Mizoue T, Tokui N, et al. Prospective study of diabetes mellitus and liver cancer in Japan. *Diabetes Metab Res Rev* 2001; 17: 374-379.

31. El-Serag HB, Tran T, Everhart JE. Diabetes increases the risk of chronic liver disease and hepatocellular carcinoma. *Gastroenterology* 2004; 126: 460-468.

32. Yu MC, Tong MJ, Govindarajan S, Henderson BE. Nonviral risk factors for hepatocellular carcinoma in a low-risk population, the non-Asians of Los Angeles County, California. *J Natl Cancer Inst* 1991; 83: 1820-1826.

33. Magee C. Kidney disease and death from cancer. *Am J Kidney Dis* 2014; 63: 7-9.

34. Izzedine H, Perazella MA. Onco-nephrology: an appraisal of the cancer and chronic kidney disease links. *Nephrol Dial Transplant* 2015; 30: 1979-1988.

35. Bruix J, Sherman M, Practice Guidelines Committee AAftSoLD. Management of hepatocellular carcinoma. *Hepatology* 2005; 42: 1208-1236.

36. Makuuchi M, Kokudo N, Arii S, et al. Development of evidence-based clinical guidelines for the diagnosis and treatment of hepatocellular carcinoma in Japan. *Hepatol Res* 2008; 38: 37-51.

37. Llovet JM, Di Bisceglie AM, Bruix J, et al. Design and endpoints of clinical trials in hepatocellular carcinoma. *J Natl Cancer Inst* 2008; 100: 698-711.

38. Takayama T, Makuuchi M, Kojiro M, et al. Early hepatocellular carcinoma: pathology, imaging, and therapy. *Ann Surg Oncol* 2008; 15: 972-978.

39. Ye SL, Takayama T, Geschwind J, et al. Current approaches to the treatment of early hepatocellular carcinoma. *Oncologist* 2010; 15 Suppl 4: 34-41.

40. Kuo MJ, Chen HH, Chen CL, et al. Cost-effectiveness analysis of population-based screening of hepatocellular carcinoma: Comparing ultrasonography with two-stage screening. *World J Gastroenterol* 2016; 22: 3460-3470.

41. Bertakis KD, Azari R, Helms LJ, et al. Gender differences in the utilization of health care services. *J Fam Pract* 2000; 49: 147-152.

For peer review only - http://bmjopen.bmj.com/site/about/guidelines.xhtml

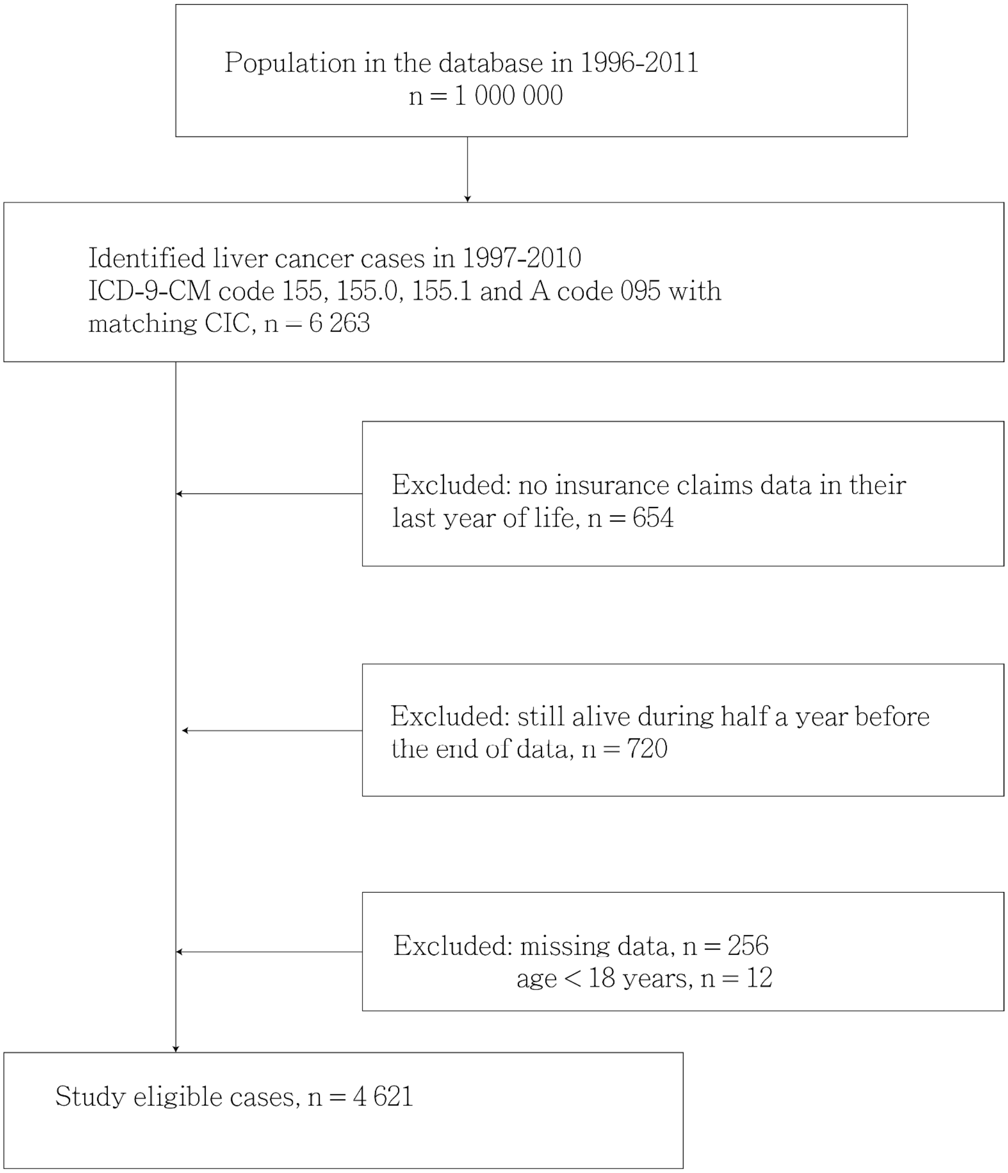

467x543mm (300 x 300 DPI)

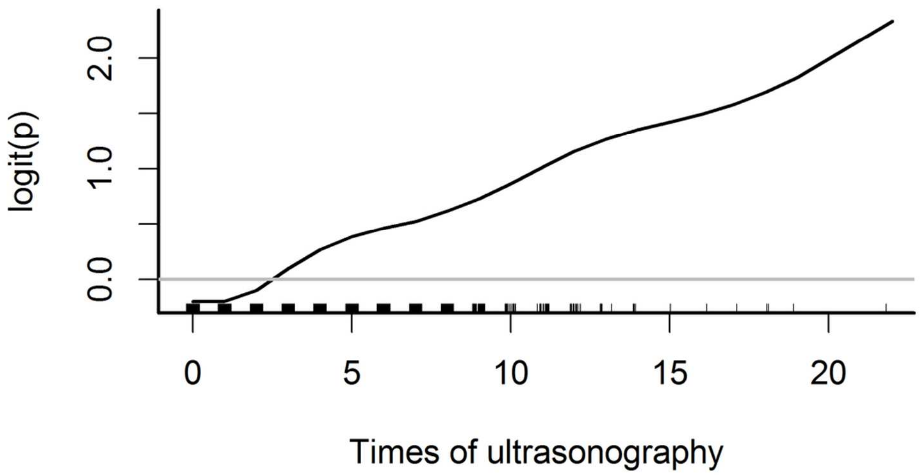

101x67mm (300 x 300 DPI)

view only

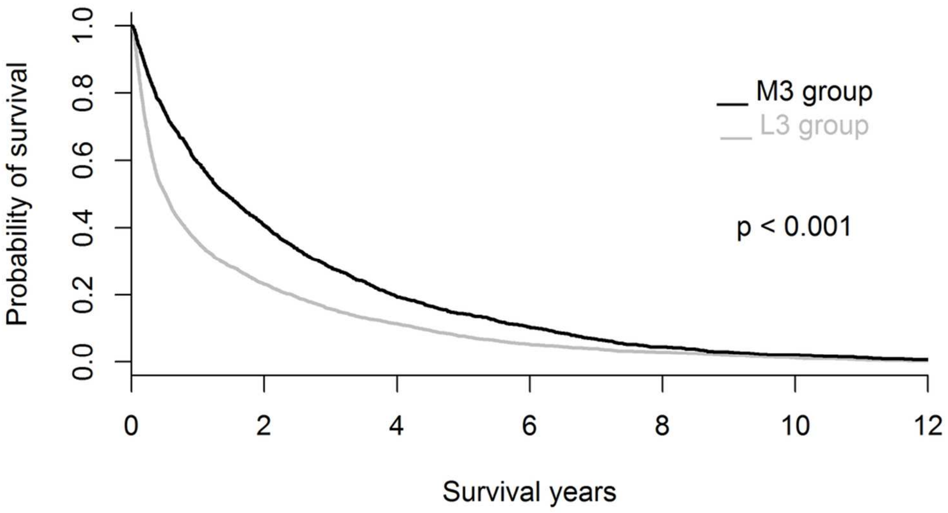

101x67mm (300 x 300 DPI)

view only

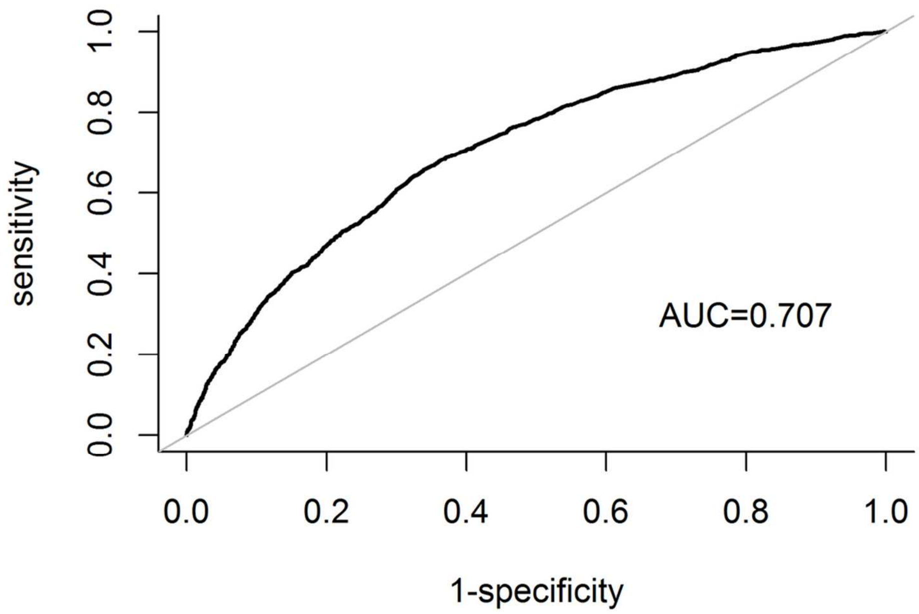

101x81mm (300 x 300 DPI)

For peer review only

STROBE Statement—Checklist of items that should be included in reports of ***cohort studies***

	Item No	Recommendation
**Title and abstract**	1	(*a*) Indicate the study's design with a commonly used term in the title or the abstract (page 2)
		(*b*) Provide in the abstract an informative and balanced summary of what was done and what was found (page 2)
**Introduction**		
Background/rationale	2	Explain the scientific background and rationale for the investigation being reported (page 5-6)
Objectives	3	State specific objectives, including any prespecified hypotheses (page 6)
**Methods**		
Study design	4	Present key elements of study design early in the paper (page 6)
Setting	5	Describe the setting, locations, and relevant dates, including periods of recruitment, exposure, follow-up, and data collection (page 6)
Participants	6	(*a*) Give the eligibility criteria, and the sources and methods of selection of participants. Describe methods of follow-up (page 7)
		(*b*) For matched studies, give matching criteria and number of exposed and unexposed (not indicated)
Variables	7	Clearly define all outcomes, exposures, predictors, potential confounders, and effect modifiers. Give diagnostic criteria, if applicable (page 7-8)
Data sources/ measurement	8*	For each variable of interest, give sources of data and details of methods of assessment (measurement). Describe comparability of assessment methods if there is more than one group (page 7-9)
Bias	9	Describe any efforts to address potential sources of bias (page 7)
Study size	10	Explain how the study size was arrived at (page 7)
Quantitative variables	11	Explain how quantitative variables were handled in the analyses. If applicable, describe which groupings were chosen and why (page 8)
Statistical methods	12	(*a*) Describe all statistical methods, including those used to control for confounding (page 9)
		(*b*) Describe any methods used to examine subgroups and interactions (page 9)
		(*c*) Explain how missing data were addressed (Page 7, figure 1)
		(*d*) If applicable, explain how loss to follow-up was addressed (page 7, figure 1)
		(*e*) Describe any sensitivity analyses (page 10)
**Results**		
Participants	13*	(a) Report numbers of individuals at each stage of study—eg numbers potentially eligible, examined for eligibility, confirmed eligible, included in the study, completing follow-up, and analysed (page 10)
		(b) Give reasons for non-participation at each stage (page 10, figure 1)
		(c) Consider use of a flow diagram (page 10, figure 1)
Descriptive data	14*	(a) Give characteristics of study participants (eg demographic, clinical, social) and information on exposures and potential confounders (page 11)
		(b) Indicate number of participants with missing data for each variable of interest (page 10, figure 1)
		(c) Summarise follow-up time (eg, average and total amount) (page 10)
Outcome data	15*	Report numbers of outcome events or summary measures over time (page 11)
Main results	16	(*a*) Give unadjusted estimates and, if applicable, confounder-adjusted estimates and their precision (eg, 95% confidence interval). Make clear which confounders were

For peer review only

For peer review only - http://bmjopen.bmj.com/site/about/guidelines.xhtml

		adjusted for and why they were included (page 11)
		(*b*) Report category boundaries when continuous variables were categorized (page 11)
		(*c*) If relevant, consider translating estimates of relative risk into absolute risk for a meaningful time period (page 13)
Other analyses	17	Report other analyses done—eg analyses of subgroups and interactions, and sensitivity analyses (page 13)
**Discussion**		
Key results	18	Summarise key results with reference to study objectives (page 13)
Limitations	19	Discuss limitations of the study, taking into account sources of potential bias or imprecision. Discuss both direction and magnitude of any potential bias (page 17)
Interpretation	20	Give a cautious overall interpretation of results considering objectives, limitations, multiplicity of analyses, results from similar studies, and other relevant evidence (page 13-14)
Generalisability	21	Discuss the generalisability (external validity) of the study results (page 14)
**Other information**		
Funding	22	Give the source of funding and the role of the funders for the present study and, if applicable, for the original study on which the present article is based (page 18)

*Give information separately for exposed and unexposed groups.

Note: An Explanation and Elaboration article discusses each checklist item and gives methodological background and published examples of transparent reporting. The STROBE checklist is best used in conjunction with this article (freely available on the Web sites of PLoS Medicine at http://www.plosmedicine.org/, Annals of Internal Medicine at http://www.annals.org/, and Epidemiology at http://www.epidem.com/). Information on the STROBE Initiative is available at http://www.strobe-statement.org.

For peer review only

For peer review only - http://bmjopen.bmj.com/site/about/guidelines.xhtml

BMJ Open

Effect of Ultrasonography Surveillance on Survival of Patients with Liver Cancer: a Population-Based Longitudinal Study

Journal:	*BMJ Open*
Manuscript ID	bmjopen-2017-015936.R1
Article Type:	Research
Date Submitted by the Author:	10-May-2017
Complete List of Authors:	Chiang, Jui-Kun<br>Lin , Chih-Wen<br>Kao, Yee-Hsin; Family Medicine; Tainan Municipal Hospital
**Primary Subject Heading**:	General practice / Family practice
Secondary Subject Heading:	Gastroenterology and hepatology, Epidemiology
Keywords:	PREVENTIVE MEDICINE, Hepatobiliary disease < GASTROENTEROLOGY, Hepatobiliary tumours < ONCOLOGY, PRIMARY CARE

SCHOLARONE™
Manuscripts

For peer review only

Effect of Ultrasonography Surveillance on Survival of Patients with Liver Cancer: A Population-Based Longitudinal Study

Jui-Kun Chiang[1], M.D., MSc, Lin Chih-Wen[2, 3] M.D., MBA, Yee-Hsin Kao[4], M.D.

[1]Department of Family Medicine, Dalin Tzu Chi Hospital, Buddhist Tzu Chi Medical Foundation, Dalin, Chiayi, Taiwan

[2]Department of Medical Imaging, Dalin Tzu Chi Hospital, Buddhist Tzu Chi Medical Foundation, Dalin, Chiayi, Taiwan.

[3]School of Medicine, Tzu Chi University, Hualien, Taiwan.

[4]Department of Family Medicine, Tainan Municipal Hospital, Tainan, Taiwan.

For peer review only

Running head: ultrasonography surveillance on survival for liver cancer patient

Corresponding author: Yee-Hsin Kao: m2200767@gmail.com
Department of Family Medicine
Tainan Municipal Hospital
670 Chung-Te Road
Tainan 701, Taiwan
E-mail: m2200767@gmail.com
Tel: +886 6 260-9926 × 22226
Fax: +886 6 260-9900

E-mail for Jui-Kun Chiang: jkch68@gmail.com
E-mail for Lin Chih-Wen: dalinchiayi@gmail.com

Number of manuscript Pages:
Number of Figures: 4
Number of Tables: 5
Words counts: Abstract: 246, Text: 3381 (introduction: 502, discussion: 1192)
Number of References: 53

For peer review only - http://bmjopen.bmj.com/site/about/guidelines.xhtml

Abstract

Objective Liver cancer is a growing global public health problem. Ultrasonography is an imaging tool widely used for the early diagnosis of liver cancer. However, the effect of ultrasonography surveillance (US) on the survival of patients with liver cancer is unknown. This study aimed to examine the association between survival and US frequency during the 2 years preceding liver cancer diagnosis.

Methods This population-based longitudinal study was conducted in Taiwan, a region with high liver cancer incidence, by using the National Health Insurance Research Database. We compared survival between patients receiving US three times or more (≥3 group) and those receiving US less than three times (<3 group) during the 2 years preceding liver cancer diagnosis. The significant factors for the ≥3 group were identified.

Results This study enrolled 4621 patients with liver cancer who died during 1997–2010. The median survival was higher in the ≥3 group (1.42 years) than in the <3 group (0.51 years). Five-year survival probability was significantly higher in the ≥3 group (14.4%) than in the <3 group (7.7%). From the multivariate logistic regression model, for the ≥3 group, the most three significant positive factors were the US indications of viral hepatitis, gallbladder diseases, and kidney urinary bladder diseases, and the significant negative factors were the male sex and the US indication of abdominal pain.

For peer review only - http://bmjopen.bmj.com/site/about/guidelines.xhtml

Conclusion Patients with liver cancer receiving US three times or more during the 2 years preceding liver cancer diagnosis might exhibit a higher 5-year survival probability.

Key words: ultrasonography surveillance (US), liver cancer, survival

Strengths and limitations of this study

A nationwide population-based study was conducted in Taiwan, a high incidence of liver cancer.

We found that the five-year survival probability was significantly higher in those who revived three times or more ultrasonography surveillance (14.4%) than those who received US less than three times (7.7%).

Ultrasonography surveillance three times or more within 2 years may be suggested to general population in the area of high incidence of liver cancer.

No information on cancer stage is available in the dataset, and some crucial potential confounding variables are also not available in the dataset for any statistical adjustment.

For peer review only - http://bmjopen.bmj.com/site/about/guidelines.xhtml

Introduction

Liver cancer is a growing global public health problem and is the fifth and ninth most common cancer in men and women worldwide, respectively, and because of its poor outcome, it is the second most common cause of cancer-related mortality, accounting for nearly 746,000 deaths worldwide in 2012.[1] Eastern and Southeastern Asia, including Taiwan, are regions with a high incidence of liver cancer. In Taiwan, liver cancer has been the second leading cause of cancer-related mortality since 2004 and accounted for 8,116 (18.6%) of 43,665 cancer deaths in 2012.[2] The known risk factors for liver cancer are viral infection (hepatitis B virus [HBV] and hepatitis C virus [HCV] infection), alcohol consumption and Alfa toxins, diabetes, and metabolic (nonalcoholic fatty liver disease and hereditary hemochromatosis) and immune-related (primary biliary cirrhosis and autoimmune hepatitis) diseases.[3] A previous study reported that chronic HBV and HCV infections are the major risk factors for liver cancer.[4] For effective control and treatment of viral hepatitis, two programs have been implemented in Taiwan. The mass vaccination program against HBV has considerably reduced the HBV carrier rate in children and adolescents and consequently reduced the incidence of childhood liver cancer in Taiwan.[5-8] The other program is the pilot program titled “Strengthening of treatment for chronic hepatitis B and C under the National Health Insurance” initiated in October 2003 by the

For peer review only - http://bmjopen.bmj.com/site/about/guidelines.xhtml

Taiwanese Center for Disease Control. The program has made attempts to ensure that patients with chronic hepatitis B and C are registered, so that they can benefit from appropriate diagnosis, monitoring, and treatment.[9] Accordingly, the incidence of liver cancer significantly decreased by an average annual percentage change of 1.1% from 2002 to 2012, and the 5-year relative survival rates for liver cancer were 52.0% for stage I, 2.9% for stage IV, and 28.9% for all stages.[10]

The diagnosis of hepatocellular cancer is based on a combination of radiologic, serologic, and histopathologic criteria. Studies have reported that alpha-fetoprotein determination lacks adequate sensitivity and specificity for effective surveillance.[11 12] Thus, surveillance must be based on ultrasonography, and the recommended screening interval is 6 months.[13 14] A previous study reported that for the detecting early-stage liver cancer, ultrasonography had a sensitivity of approximately 63% and a high specificity of approximately 85–90%.[12] Moreover, ultrasonography is a noninvasive and safe method, is well accepted by patients, and is relatively inexpensive.

Previous studies have reported that most patients with hepatocellular carcinoma exhibit low survival. However, patients with early-stage hepatocellular carcinoma who receive potentially curative therapy exhibit considerably improved survival (5-year survival = 40%–70%).[15] A systemic review provided very-low-strength evidence about the effects of hepatocellular carcinoma screening on mortality in

For peer review only - http://bmjopen.bmj.com/site/about/guidelines.xhtml

patients with chronic hepatitis.[16] Regular surveillance with abdominal ultrasonography might facilitate the early detection of liver cancer, thus improving the survival of at-risk patients. This study examined the effect of ultrasonography surveillance (US) on survival and the association between survival and the frequency of ultrasonography during the 2 years preceding liver cancer diagnosis and identified the predictors of receiving more US preceding liver cancer diagnosis.

Methods

Data source and patient identification

In this nationwide population-based study, we analyzed the data from the National Health Insurance Research Database (NHIRD) of Taiwan. The NHI program, established in 1995, is a single-payer health insurance system that covered up to 99.9% of all residents of Taiwan in 2012. Moreover, 97% of medical providers nationwide are affiliated with the program.[17] The Longitudinal Health Insurance Database (LHID) is a nationwide representative database containing all original claims data for the period of 1996–2011 for one million NHI beneficiaries randomly sampled from the 23.22 million NHI enrollees. The availability of such a population-based database has stimulated and facilitated academic research in various scientific disciplines, particularly in the field of health research.[18] The accuracy of

For peer review only - http://bmjopen.bmj.com/site/about/guidelines.xhtml

diagnoses registered in the NHIRD has been validated using five-digit International Classification of Diseases, Ninth Revision, Clinical Modification (ICD-9-CM) codes, including those for diabetes and cancer, indicating the reliability of the data source.[19] In Taiwan, all cancer patients are designated as having a catastrophic illness. We identified liver cancer patients with catastrophic illness designations in LHID2000 and followed up these patients until December 2011. The ICD-9-CM and A codes were used to define liver cancer (155, 155.0, 155.1, A095).

Study sample

This study included patients aged ≥18 years with a first-time diagnosis of liver cancer between January 1, 1997, and December 31, 2010. The NHIRD was used to identify patients with liver cancer, and the cancer cases were confirmed using the Registry of Catastrophic Illness. Patients were excluded if they made no insurance claims during their final year of life, were still alive during half a year before the end of the dataset, or had missing data. A total of 4621 patients with liver cancer were enrolled in this study (Figure 1).

Definition of frequency of abdominal ultrasonography surveillance during 2 years preceding liver cancer diagnosis

We classified the study population into two groups based on to the frequency of

For peer review only - http://bmjopen.bmj.com/site/about/guidelines.xhtml

US during the 2 years preceding liver cancer diagnosis: <3 group (patients with liver cancer who received US less than three times; n = 3149) and ≥3 group (those who revived regular US, i.e., three times or more; n = 1472).

We classified the diseases that are the indications for ultrasonography into seven different groups by using the ICD-9-CM diagnosis codes. The seven disease groups were as follows: (1) liver-related diseases including chronic hepatitis, cirrhosis, and other liver disorders (ICD-9 codes: 571, 571.x, 572, 5720, 573, and 573.x); (2) viral hepatitis infection (ICD-9 codes for HBV: V0261, 070.20–070.23, and 070.30–070.33; for HCV: V0262, 070.41, 070.44, 070.51, and 070.54); (3) gallbladder and biliary tract-related diseases (ICD-9 codes: 574–576); (4) abdominal pain conditions (ICD-9 codes: 789.0x, 789.4x, 789.6x, and 789.9); (5) kidney–urinary–bladder (KUB) diseases (ICD-9 codes: 591, 592, 592.0, 592.1, 592.9, 593, and 593.2); (6) pancreatic diseases (ICD-9 code: 577.x); and (7) other conditions such as hepatomegaly, splenomegaly, abdominal or pelvic mass, and ascites (ICD-9 codes: 7891, 7892, 7893x,and 7895).

Definition of variables

Charlson comorbidity index (CCI) was calculated by examining ICD-9-CM diagnosis and procedure codes recorded in the year before diagnosis, according to the Deyo method, and CCI was applied to inpatient and outpatient claims, according to

For peer review only - http://bmjopen.bmj.com/site/about/guidelines.xhtml

the method of Klabundle *et al.*[20-22]

The study protocol was reviewed and approved by the Research Ethics Committee of Dalin Tzu Chi Hospital, Buddhist Tzu Chi Medical Foundation, Taiwan (Approval No. B10304018). Because the NHIRD contains only deidentified secondary data, the review board waived the requirement for informed consent.

Statistical analyses

Continuous variables are expressed in mean ± standard deviation (SD), and categorical variables are presented in frequency and percentage. In univariate analysis, two-sample *t* test, Wilcoxon rank-sum test, chi-square test, and Fisher's exact test were used to examine the differences in the distributions of continuous variables and categorical variables between the two groups. The survival duration (years) was defined as the duration from the day of diagnosis to the day of death. Generalized additive models (GAMs) were fitted to detect the potential nonlinear effects of continuous covariates and to identify the appropriate cutoff points for discretizing continuous covariates, if necessary, during the stepwise variable selection. Computationally, the VGAM function (with the default values of smoothing parameters) of the VGAM package was used to fit GAMs for the binary outcome in R.

Cox proportional hazards regression models were used to estimate the

For peer review only

For peer review only - http://bmjopen.bmj.com/site/about/guidelines.xhtml

event-specific hazard ratios (HRs) and 95% confidence intervals (CIs) of all the variables listed in Tables 1 and 2. Subsequently, multivariate analysis was conducted by fitting multiple logistic regression models with the stepwise variable selection procedure to identify the crucial predictors of receiving US three times or more during the 2 years preceding liver cancer diagnosis. The goodness-of-fit (GOF) of the final logistic regression model was assessed by estimating area under the receiver operating characteristic (ROC) curve (AUC; also called the c statistic), where $0 \leq c \leq 1$, and the Hosmer–Lemeshow GOF test. In practice, $c \geq 0.7$ suggests an acceptable level of discrimination power for a fitted logistic regression model. Moreover, in the Hosmer–Lemeshow test, $p > 0.05$ indicates a good fit for the logistic regression model. The variance inflating factor of ≥10 for continuous covariates or ≥2.5 for categorical covariates indicate the occurrence the multicollinearity problem among some covariates in the fitted logistic regression model. Statistical analysis was performed using the R 3.0.2 software (R Foundation for Statistical Computing, Vienna, Austria). Two-sided $p \leq 0.05$ was considered significant.

Results

We enrolled 4621 adult patients (3287 men and 1334 women; ratio = 2.46:1) with liver cancer who died during 1997–2010. Figure 1 depicts the study design. We

For peer review only - http://bmjopen.bmj.com/site/about/guidelines.xhtml

explored the association between the frequency of ultrasonography during the 2 years preceding liver cancer diagnosis and 5-year survival probability. The optimal cutoff point for receiving US during the 2 years preceding liver cancer diagnosis was set at three times for higher 5-year survival probability (Figure 2).

Table 1 summarizes of the demographic characteristics of the study patients and indicates that the patients in the ≥3 group were more likely to be female ($p < 0.001$) and were more likely to have the comorbidities of viral hepatitis (HBV or HCV infection) ($p < 0.001$), cirrhosis ($p < 0.001$), diabetes ($p < 0.001$), hypertension ($p < 0.001$), and stroke ($p = 0.001$). The median survival of the ≥3 group (1.42 years) was higher than that of the <3 group (0.51 years) ($p < 0.001$). Table 2 summarizes the indications for abdominal US used in this study. The most frequent indications were viral hepatitis (2580, 55.8%), gallbladder diseases (1690, 36.6%), and abdominal pain (1387, 30.0%). In this study, all indications, except for abdominal pain ($p = 0.098$), were significantly higher in the ≥3 group than in the L3 group. Compared with the <3 group, significantly higher proportions of the ≥3 group received all treatments for liver cancer, except for radiotherapy ($p = 0.097$), and the ≥3 group had significantly higher 5-year survival probability (7.7% and 14.4%, respectively; $p < 0.001$) (Table 3). From the multivariate Cox proportional hazards regression model, the positive significant predictors of the death event were the male sex (HR: 1.14, 95% CI:

For peer review only - http://bmjopen.bmj.com/site/about/guidelines.xhtml

1.07–1.22), age (per 5 years) (HR: 1.02, 95% CI: 1.01–1.03), diabetes (HR: 1.13, 95% CI: 1.04–1.22), and high CCI score (per the score of 1) (HR: 1.07, 95% CI: 1.05–1.08). Moreover, the negative significant predictors of the death event were hypertension (HR: 0.83, 95% CI: 0.78–0.89), stroke (HR: 0.78, 95% CI: 0.72–0.84), chronic kidney diseases (CKDs) (HR: 0.84, 95% CI: 0.74–0.92), viral hepatitis (HBV or HCV infection) (HR: 0.79, 95% CI: 0.72–0.88), receiving US three times or more during the 2 years preceding liver cancer diagnosis (HR: 0.80, 95% CI: 0.75-0.85). The significant indications for US included KUB diseases (HR: 0.92, 95% CI: 0.86–0.995), gallbladder diseases (HR: 0.90, 95% CI: 0.85–0.96), chronic hepatitis (except for HBV or HCV infection) (HR: 0.90, 95% CI: 0.81–0.999), and pancreatic diseases (HR: 0.80, 95% CI: 0.71–0.91). Moreover, the significant complications of liver cancer were upper gastrointestinal bleeding (gastric ulcer or duodenal ulcer) (HR: 0.92, 95% CI: 0.86–0.98) and esophageal variceal bleeding (HR: 0.90, 95% CI: 0.83–0.97), and the significant treatments received for liver cancer were transcatheter arterial chemoembolization (TACE) (HR: 0.82, 95% CI: 0.76–0.89), radiotherapy (HR: 0.81, 95% CI: 0.75–0.87), radiofrequency ablation (RFA) (HR: 0.79, 95% CI: 0.68–0.93), chemotherapy (HR: 0.71, 95% CI: 0.67–0.76), resection (HR: 0.67, 95% CI: 0.61–0.73), and percutaneous ethanol injection (PEI) (HR: 0.64, 95% CI: 0.57–0.70). The significant demographic factors were employment (HR: 0.89, 95% CI:

For peer review only - http://bmjopen.bmj.com/site/about/guidelines.xhtml

0.83-0.95) and living in Northern Taiwan (HR: 0.91, 95% CI: 0.85-0.97) (Table 4). Survival probability was significantly higher in the ≥3 group than in the <3 group ($p <$ 0.001; Figure 3). From the multivariate logistic regression model, the significant positive factors for the ≥3 group were viral hepatitis (HBV or HCV infection) (odds ratio [OR]: 3.66, 95% CI: 2.76–4.85), gallbladder diseases (OR: 1.82, 95% CI: 1.59–2.09), KUB diseases (OR: 1.80, 95% CI: 1.53–2.11), pancreatic diseases (OR: 1.79, 95% CI: 1.37–2.34), chronic hepatitis (except for viral hepatitis; OR: 1.75, 95% CI: 1.31–2.34), CKD (OR: 1.60, 95% CI: 1.30–1.96), diabetes (OR: 1.56, 95% CI: 1.31–1.86), the US indication of other conditions (OR: 1.47, 95% CI: 1.29-1.69), hypertension (OR: 1.17, 95% CI: 1.01–1.34), living in Central Taiwan (OR: 1.16, 95% CI: 1.01–1.34). Moreover, the significant negative factors were the male sex (OR: 0.60, 95% CI: 0.52–0.69) and the US indication of abdominal pain (OR: 0.81, 95% CI: 0.70–0.93) (Table 5). In the Hosmer–Lemeshow test, the p value of 0.156 indicated a good fit, and the AUC was acceptable (0.707, 95% CI: 0691–0723) (Figure 4).

Discussion

In this study, we found that patients with liver cancer who received US three times or more during the 2 years preceding liver cancer diagnosis exhibited high 5-year survival, (14.4% vs. 7.7%). We also found that compared with the <3 group, higher proportions of the ≥3 group received treatment for early stage of liver cancer,

For peer review only - http://bmjopen.bmj.com/site/about/guidelines.xhtml

such as PEI, hepatectomy, RFA, and liver transplantation. There was a linear dose-response relationship between US frequency and logit of 5-year survival probability, as shown in the Figure 2. A similar result was also reported in previous study.[23] Further cost-effectiveness analyses are needed to justify the recommendation of ultrasonography surveillance three times or more within 2 years to high risk people or general population in the area of high incidence of liver cancer.

One previous trial also reported a similar result, in which biannual US for patients with hepatitis B virus infection reduced hepatocellular carcinoma mortality by 37%.[24] Another cohort study reported that patients with viral hepatitis who received routine US exhibited higher 5-year survival than those who did not (31.84% and 20.67%, respectively).[25] Some previous articles also reported that certain populations at a high-risk for developing HCC will benefit from more intensive US surveillance.[26-30] Moreover, a trial reported that early-stage hepatocellular carcinoma was more likely to be detected in patients with viral hepatitis receiving US at a 4-month interval than in those receiving US at a 12-month interval; however, the overall survival was not different at the 4-year follow-up.[31] A systemic review reported that the effects of hepatocellular carcinoma screening on mortality in patients with chronic hepatitis were uncertain.[16] Thus, receiving US at a high frequency had no beneficial effect on the 5-year survival probability for patients with hepatocellular

For peer review only - http://bmjopen.bmj.com/site/about/guidelines.xhtml

carcinoma. This finding may be because the early detection of liver cancer improves the rate of curative treatments. However, the further, regular follow-up for possible recurrences and treatments is vital. The prognosis of patients with liver cancer is not solely related to the tumor stage.[32] Although the survival of patients with liver cancer has improved in the past decade, physicians caring for at-risk patients need to provide regular high-quality screening, including ultrasonography.[13]

Although advanced diagnostic techniques such as dynamic multiphasic multidetector-row computed tomography and magnetic resonance imaging are the standard diagnostic methods for the noninvasive diagnosis of liver cancer, ultrasonography still plays a crucial role in liver cancer surveillance.[33] The recommended interval for US screening is 6 months for patients with viral hepatitis infection and 3 months for patients with cirrhosis in Taiwan. Ultrasonography can be performed in hospital and clinics, if indicated, and the cost of each ultrasonography screening (USD $23.1) is covered by the NHI program in Taiwan.

In this study, we found that patients with chronic hepatitis (HBV, HCV infection, and non-B or non-C hepatitis), diabetes, CKD, cerebral vascular accident (CVA), hypertension, gallbladder diseases, KUB diseases, pancreatic diseases, and other conditions (i.e., hepatomegaly, splenomegaly, abdominal or pelvic mass, and ascites) were more likely to receive US three times or more during the 2 years preceding liver

For peer review only - http://bmjopen.bmj.com/site/about/guidelines.xhtml

cancer diagnosis. One reason that patients with diabetes were more likely to receive US three times or more in this study might be that these patients are at a high risk of primary liver cancer,[34-36] and the association between diabetes and liver cancer is significantly strengthened when the history of diabetes is longer than 10 years.[37 38] Data suggest that insulin might enhance and certain oral glucose-lowering medications and statins possibly decrease the risk of HCC.[39 40] One reason for those patients with CKD were more likely to receive US three times or more in this study might be the close link between the kidney and cancer,[41] and cancer has become increasingly recognized as a complication and a major cause of morbidity and mortality in the CKD population.[42] The reason that patients with comorbidities such as CVA, hypertension, gallbladder diseases, KUB diseases, pancreatic diseases, and other conditions were more likely to receive US three times or more in this study might be that patients with more comorbidities and physician visits probably undergo more laboratory tests and imaging studies, including US, if they experience any discomfort. Diabetes, hypertension, obesity, metabolic syndrome, mixed hyperlipidemia and hypocholesterolemia due to familial hypobetalipoproteinemia were the non-alcoholic fatty liver disease risk.[43] Non-alcoholic fatty liver disease has an etiology role in cryptogenic cirrhosis that is associated with a poor prognosis and high risk of cardiovascular disease and early HCC development.[44 45] In this study, the

For peer review only - http://bmjopen.bmj.com/site/about/guidelines.xhtml

non-alcoholic fatty liver was included in group of liver-related diseases including chronic hepatitis, cirrhosis, and other liver disorders. The precise classification for non-alcoholic fatty liver was also one of the limitations of this study originated from claim data. In this study, we classified the diseases that are the indications for ultrasonography as seven diseases groups. Patients with above indications could receive US surveillance due to US surveillance was easily available in Taiwan. The underlying cause of pancreatic disease included alcohol abuse, biliary disease, hyperlipidemia, and other risk factors, etiologies or idiopathic disease.[46] It is another limitation in this study, the baseline data were not recorded in the claim data for investigate.

In this study, compared with the <3 group, higher proportions of the ≥3 group received treatments for early-stage liver cancer, such as PEI, hepatectomy, RFA, and liver transplantation. These treatments were reported as therapies for early-stage hepatocellular carcinoma in previous studies.[47-50] We also found that compared with the <3 group, higher proportions of the ≥3 group received TACE, although the therapeutic effects of TACE for early-stage hepatocellular carcinoma are unclear .[51]

A previous study reported that mass screening for liver cancer by using US in high-endemic regions, including Taiwan, is cost-effective and recommended.[52] In this study, the significant positive factors for the ≥3 group were viral hepatitis, gallbladder

For peer review only

For peer review only - http://bmjopen.bmj.com/site/about/guidelines.xhtml

diseases, KUB diseases, pancreatic diseases, chronic hepatitis (except for viral infection), diabetes, other indications for US (hepatomegaly, splenomegaly, etc.), hypertension, and living in Central Taiwan. However, the significant negative factors for the ≥3 group were the male sex and the US indication of abdominal pain. The reason might be that men have fewer intentions for medical service than women.[53] The US indication of abdominal pain might be a significant negative factor because after seeking medical service for one event and after solving the problem, patients do not return for subsequent follow-up. However, the precise reason for this indication is not available from the claims data, and this is a limitation of this study. Although such patients more likely died of liver cancer, the cause of death was not recorded in claim data, and thus it was one of the limitations in this study. This was an observational cohort study, and thus its validity might be jeopardized by confounding biases of unmeasured or unknown covariates. Hence, if it is feasible, randomized clinical trials should be conducted before its conclusions are applied to clinical practice in Taiwan and elsewhere. Other limitations of this study are that no information on cancer stage is available in the dataset, and some crucial potential confounding variables, such as body mass index, smoking, and alcohol intake, are also not available in the dataset for any statistical adjustment.

Conclusions

For peer review only - http://bmjopen.bmj.com/site/about/guidelines.xhtml

Five-year survival probability might improve in patients with liver cancer who receive abdominal US three times or more during the 2 years preceding liver cancer diagnosis. Additional studies should prospectively investigate whether abdominal US can be used to identify early-stage liver cancer and then examine whether further treatment with regular follow-up improves survival.

Acknowledgements

This study is based in part on data from the National Health Insurance Research Database provided by the Bureau of National Health Insurance, Department of Health and managed by the National Health Research Institutes. The interpretation and conclusions contained herein do not represent those of the Bureau of National Health Insurance, Department of Health or National Health Research Institutes.

Footnotes

Contributors: JKC, LCW and YHK designed, conducted and drafted the manuscript. JKC analyzed the data. All authors contributed to the manuscript, revised drafts critically for important intellectual content, and read and approved the final manuscript.

Funding: JK Chiang received research grants from Dalin Tzu Chi Hospital, Buddhist Tzu Chi Medical Foundation (DTCRD105 (2)-E-22). The funding source had no

For peer review only - http://bmjopen.bmj.com/site/about/guidelines.xhtml

involvement in study design; in the collection, analysis and interpretation of data; in the writing of the report; and in the decision to submit the article for publication.

Competing interests: None declared.

Ethics approval: The study protocol was reviewed and approved by the Research Ethics Committee of Dalin Tzu Chi Hospital, Buddhist Tzu Chi Medical Foundation, Taiwan (Approval No. B10304018).

Data sharing statement: No additional data are available.

For peer review only - http://bmjopen.bmj.com/site/about/guidelines.xhtml

Table 1. Comparison of demographic characteristics between the <3 group and the ≥3 group

Variable	<3 group, n (%)	≥3 group, n (%)	*p* value
Total number (%)	3149 (68.1)	1472 (31.9)	
Age (years)	63.04 ± 13.92	63.77 ± 11.76	0.474
Gender			< 0.001
female	807 (25.6)	527 (35.8)	
male	2342 (74.4)	945 (64.2)	
Survival*	0.51 (0.17, 1.81)	1.42 (0.48, 3.34)	< 0.001
HBV	1119 (35.5)	619 (42.1)	< 0.001
HCV	660 (21.0)	620 (42.1)	< 0.001
Diabetes	535 (17.0)	361 (24.5)	< 0.001
CKD	276 (8.8)	232 (15.8)	< 0.001
CVA	535 (17.0)	297 (20.2)	0.001
Hypertension	1173 (37.2)	691 (46.9)	< 0.001
Cirrhosis	2048 (65.0)	1183 (80.4)	< 0.001
Employment (yes)	1873 (59.5)	891 (60.5)	0.499
Northern area in Taiwan	1063 (33.8)	467 (31.7)	0.178
Central area in Taiwan	888 (28.2)	461 (31.3)	0.031
Southern area in Taiwan	1032 (32.8)	480 (32.6)	0.920
Eastern area in Taiwan	153 (4.9)	57 (3.9)	0.150

Abbreviations: HBV, hepatitis B virus; HCV, hepatitis C virus; CKD, chronic kidney disease; CVA, cerebral vascular accident; CCI: Charlson comorbidity index

Survival*: median (first quartile, third quartile)

For peer review only - http://bmjopen.bmj.com/site/about/guidelines.xhtml

Table 2. Ultrasonography surveillance indications in the <3 group and the ≥3 group

Variable	Total number (%)	<3 group, n (%)	≥3 group, n (%)	*p* value
Viral hepatitis (HBV, HCV)	2580 (55.8)	1556 (49.4)	1024 (69.6)	< 0.001
Chronic hepatitis (except HBV, HCV)	1560 (33.8)	1179 (37.4)	381 (25.9)	< 0.001
Gall bladder diseases	1690 (36.6)	989 (31.4)	701 (47.6)	< 0.001
Abdominal pain	1387 (30.0)	921 (29.2)	466 (31.7)	0.098
KUB diseases	928 (20.1)	524 (16.6)	404 (27.4)	< 0.001
Pancreas diseases	271 (5.9)	133 (4.2)	138 (9.4)	< 0.001
Others*	1780 (38.5)	1085 (34.5)	695 (47.2)	< 0.001

Abbreviations: HBV, hepatitis B virus; HCV, hepatitis C virus; KUB, kidney–urinary–bladder

Others* include hepatomegaly, splenomegaly, abdominal or pelvic mass, and ascites.

For peer review only - http://bmjopen.bmj.com/site/about/guidelines.xhtml

Table 3. Comparison of treatments for ultrasonography surveillance between the <3 group and the ≥3 group

Variable	<3 group, n (%)	≥3 group, n (%)	*p* value
PEI	200 (6.4)	244 (16.6)	< 0.001
Hepatectomy	343 (10.9)	221 (15.0)	< 0.001
RFA	71 (2.3)	101 (6.9)	< 0.001
Liver transplantation	2 (0.1)	6 (0.4)	0.015
TACE	584 (18.5)	453 (30.8)	< 0.001
Chemotherapy	1169 (37.1)	677 (46.0)	< 0.001
Radiotherapy	647 (20.5)	334 (22.7)	0.097
5 years survival probability	7.7%	14.4%	< 0.001

Abbreviations: HBV, hepatitis B virus; HCV, hepatitis C virus; CKD, chronic kidney disease; CVA, cerebral vascular accident; CCI, Charlson comorbidity index; DU, duodenal ulcer; GU, gastric ulcer; EV; esophageal varices; PEI, percutaneous ethanol injection; RFA, radiofrequency ablation; TACE, transcatheter arterial chemoembolization; UGI, upper gastrointestinal tract.

For peer review only - http://bmjopen.bmj.com/site/about/guidelines.xhtml

Table 4. Multivariate Cox proportional hazards regression model of significant factors of the death event among patients with liver cancer

Variable	H.R.	95% C.I.	*p* value
Male	1.14	1.07-1.22	<0.001
Age (HCC diagnosis) per 5 years	1.02	1.01-1.03	0.002
Diabetes	1.13	1.04-1.22	0.004
Hypertension	0.83	0.78-0.89	<0.001
CVA	0.78	0.72-0.84	<0.001
CKD	0.84	0.74-0.92	<0.001
Viral hepatitis (HBV, HCV)	0.79	0.72-0.88	<0.001
CCI (per 1 score)	1.07	1.05-1.08	<0.001
US indications			
3 times or more US	0.80	0.75-0.85	<0.001
KUB diseases	0.92	0.86-0.995	0.037
Gall bladder diseases	0.90	0.85-0.96	0.001
Chronic liver diseases (except HBV, HCV)	0.90	0.81-0.999	0.047
Pancreas diseases	0.80	0.71-0.91	<0.001
Complication of liver cancer			
UGI bleeding (GU, DU)	0.92	0.86-0.98	0.009
EV bleeding	0.90	0.83-0.97	0.008
Treatments			
TACE	0.82	0.76-0.89	<0.001
Radiotherapy	0.81	0.75-0.87	<0.001
RFA	0.79	0.68-0.93	0.004
Chemotherapy	0.71	0.67-0.76	<0.001
hepatectomy	0.67	0.61-0.73	<0.001
PEI	0.64	0.57-0.70	<0.001
Background			
Employment	0.89	0.83-0.95	<0.001
Northern area in Taiwan	0.91	0.85-0.97	0.003

Abbreviations: HBV, hepatitis B virus; HCV, hepatitis C virus; CKD, chronic kidney disease; CVA, cerebral vascular accident; CCI, Charlson comorbidity index; DU, duodenal ulcer; GU, gastric ulcer; EV; esophageal varices; PEI, percutaneous ethanol injection; RFA, radiofrequency ablation; TACE, transcatheter arterial chemoembolization; UGI, upper gastrointestinal tract; US, ultrasonography surveillances

For peer review only

For peer review only - http://bmjopen.bmj.com/site/about/guidelines.xhtml

Table 5. Multivariate logistic regression model of the factors associated with receiving ultrasonography surveillance three times or more (≥3 group) during 2 years preceding liver cancer diagnosis

Variables	O.R.	95% C.I.	p value
Male	0.60	0.52-0.69	<0.001
Diabetes	1.56	1.31-1.86	<0.001
Hypertension	1.17	1.01-1.34	0.03
CKD	1.60	1.30-1.96	<0.001
US indications			
Viral hepatitis (HBV or HCV infection)	3.66	2.76-4.85	<0.001
Gall bladder diseases	1.82	1.59-2.09	<0.001
KUB diseases	1.80	1.53-2.11	<0.001
Pancreas diseases	1.79	1.37-2.34	<0.001
Chronic hepatitis except viral hepatitis	1.75	1.31-2.34	<0.001
Other*	1.47	1.29-1.69	<0.001
Abdominal pain	0.81	0.70-0.93	0.004
Central area in Taiwan	1.16	1.01-1.34	0.04

Abbreviations: HBV, hepatitis B virus; HCV, hepatitis C virus; CKD, chronic kidney disease; CVA, cerebral vascular accident; CCI: Charlson comorbidity index; KUB: kidney urinary bladder; US, ultrasonography surveillance

US indication: other* includes hepatomegaly, splenomegaly, abdominal or pelvic mass, and ascites.

For peer review only

Figure Legends

Figure 1. The study flowchart.

Figure 2. Smoothing curve of the frequency of ultrasonography during 2 years preceding liver cancer against 5-year survival probability after adjustment. (*x*-axis: frequency of ultrasonography during 2 years preceding liver cancer; *y*-axis: p, probability of 5-year survival.)

Figure 3. Survival curves stratified by two groups listed in Table 1 (less than 3 times [<3 group] and 3 times or more [≥3 group] ultrasonography surveillances during 2 years preceding liver cancer diagnosis)

Figure 4. Receiver operating characteristic curves for significant factors listed in Table 5 for predicting receiving ultrasonography three or more times during 2 years preceding liver cancer diagnosis

For peer review only - http://bmjopen.bmj.com/site/about/guidelines.xhtml

References

1. GLOBOCAN 2012: Estimated Cancer Incidence, Mortality and Prevalence Worldwide in 2012. Available at : http://globocan.iarc.fr/Pages/fact_sheets_cancer.aspx. Accessed July 6, 2016.
2. Ministry of Health and Welfare. Statistics of Causes of Death, Volume1. Available at : http://www.mohw.gov.tw/cht/DOS/Statistic.aspx?f_list_no=312&fod_list_no=2747 Accessed January 18, 2016.
3. Parikh S, Hyman D. Hepatocellular cancer: a guide for the internist. *Am J Med* 2007; 120: 194-202.
4. Gomaa AI, Khan SA, Toledano MB, et al. Hepatocellular carcinoma: epidemiology, risk factors and pathogenesis. *World J Gastroenterol* 2008; 14: 4300-4308.
5. Chen DS, Hsu NH, Sung JL, et al. A mass vaccination program in Taiwan against hepatitis B virus infection in infants of hepatitis B surface antigen-carrier mothers. *JAMA* 1987; 257: 2597-2603.
6. Liu CJ, Kao JH. Hepatitis B virus-related hepatocellular carcinoma: epidemiology and pathogenic role of viral factors. *J Chin Med Assoc* 2007; 70: 141-145.
7. Ni YH, Chang MH, Huang LM, et al. Hepatitis B virus infection in children and adolescents in a hyperendemic area: 15 years after mass hepatitis B vaccination. *Ann Intern Med* 2001; 135: 796-800.
8. Chang MH, Chen CJ, Lai MS, et al. Universal hepatitis B vaccination in Taiwan and the incidence of hepatocellular carcinoma in children. Taiwan Childhood Hepatoma Study Group. *N Engl J Med* 1997; 336: 1855-1859.
9. Wait S, Chen DS. Towards the eradication of hepatitis B in Taiwan. *Kaohsiung J Med Sci* 2012; 28: 1-9.
10. Chiang C-J, Lo W-C, Yang Y-W, et al. Incidence and survival of adult cancer patients in Taiwan, 2002-2012. J Formos Med Assoc. (2015), http://dx.doi.org/10.1016/j.jfma.2015.10.011
11. Lok AS, Sterling RK, Everhart JE, et al. Des-gamma-carboxy prothrombin and alpha-fetoprotein as biomarkers for the early detection of hepatocellular carcinoma. *Gastroenterology* 2010; 138: 493-502.
12. Singal A, Volk ML, Waljee A, et al. Meta-analysis: surveillance with ultrasound for early-stage hepatocellular carcinoma in patients with cirrhosis. *Aliment Pharmacol Ther* 2009; 30: 37-47.
13. Bruix J, Sherman M, American Association for the Study of Liver D.

For peer review only - http://bmjopen.bmj.com/site/about/guidelines.xhtml

Management of hepatocellular carcinoma: an update. *Hepatology* 2011; 53: 1020-1022.

14. EASL-EORTC clinical practice guidelines: management of hepatocellular carcinoma. *J Hepatol* 2012; 56: 908-943.

15. El-Serag HB, Davila JA. Surveillance for hepatocellular carcinoma: in whom and how? *Therap Adv Gastroenterol* 2011; 4: 5-10.

16. Kansagara D, Papak J, Pasha AS, et al. Screening for hepatocellular carcinoma in chronic liver disease: a systematic review. *Ann Intern Med* 2014; 161: 261-269.

17. National Health Insurance Research Database (NHIRD), Taiwan. http://nhird.nhri.org.tw/date_01.html Accessed May 22, 2016.

18. Chen YC YH, Wu JC, Haschler I, Chen TJ, Wetter T. Taiwan's National Health Insurance Research Database: administrative health care database as study object in bibliometrics. *Scientometrics* 2011; 86(2): 365-380.

19. Lin CC, Lai MS, Syu CY, et al. Accuracy of diabetes diagnosis in health insurance claims data in Taiwan. *J Formos Med Assoc* 2005; 104: 157-163.

20. Charlson ME, Pompei P, Ales KL, et al. A new method of classifying prognostic comorbidity in longitudinal studies: development and validation. *J Chronic Dis* 1987; 40: 373-383.

21. Deyo RA, Cherkin DC, Ciol MA. Adapting a clinical comorbidity index for use with ICD-9-CM administrative databases. *J Clin Epidemiol* 1992; 45: 613-619.

22. Klabunde CN, Potosky AL, Legler JM, et al. Development of a comorbidity index using physician claims data. *J Clin Epidemiol* 2000; 53: 1258-1267.

23. Wu CY, Hsu YC, Ho HJ, et al. Association between ultrasonography screening and mortality in patients with hepatocellular carcinoma: a nationwide cohort study. *Gut* 2016; 65: 693-701.

24. Zhang BH, Yang BH, Tang ZY. Randomized controlled trial of screening for hepatocellular carcinoma. *J Cancer Res Clin Oncol* 2004; 130: 417-422.

25. Thein HH, Campitelli MA, Yeung LT, et al. Improved Survival in Patients with Viral Hepatitis-Induced Hepatocellular Carcinoma Undergoing Recommended Abdominal Ultrasound Surveillance in Ontario: A Population-Based Retrospective Cohort Study. *PLoS One* 2015; 10: e0138907.

26. Santi V, Trevisani F, Gramenzi A et al. Semiannual surveillance is superior to annual surveillance for the detection of early hepatocellular carcinoma and patient survival. J Hepatol 2010; 53: 291-297.

27. Santi V, Buccione D, Di Micoli A et al. The changing scenario of hepatocellular carcinoma over the last two decades in Italy. J Hepatol 2012; 56: 397-405.

28. Giannini EG, Cucchetti A, Erroi V et al. Surveillance for early diagnosis of hepatocellular carcinoma: how best to do it? World J Gastroenterol 2013; 19:

For peer review only - http://bmjopen.bmj.com/site/about/guidelines.xhtml

8808-8821.
29. Del Poggio P, Olmi S, Ciccarese F et al. Factors that affect efficacy of ultrasound surveillance for early stage hepatocellular carcinoma in patients with cirrhosis. Clin Gastroenterol Hepatol 2014; 12: 1927-1933 e1922.
30. Cucchetti A, Trevisani F, Pecorelli A et al. Estimation of lead-time bias and its impact on the outcome of surveillance for the early diagnosis of hepatocellular carcinoma. J Hepatol 2014; 61: 333-341.
31. Wang JH, Chang KC, Kee KM, et al. Hepatocellular carcinoma surveillance at 4- vs. 12-month intervals for patients with chronic viral hepatitis: a randomized study in community. *Am J Gastroenterol* 2013; 108: 416-424.
32. Greten TF, Papendorf F, Bleck JS, et al. Survival rate in patients with hepatocellular carcinoma: a retrospective analysis of 389 patients. *Br J Cancer* 2005; 92: 1862-1868.
33. Lee JM, Yoon JH, Kim KW. Diagnosis of hepatocellular carcinoma: newer radiological tools. *Semin Oncol* 2012; 39: 399-409.
34. Adami HO, Chow WH, Nyren O, et al. Excess risk of primary liver cancer in patients with diabetes mellitus. *J Natl Cancer Inst* 1996; 88: 1472-1477.
35. La Vecchia C, Negri E, Decarli A, et al. Diabetes mellitus and the risk of primary liver cancer. *Int J Cancer* 1997; 73: 204-207.
36. Fujino Y, Mizoue T, Tokui N, et al. Prospective study of diabetes mellitus and liver cancer in Japan. *Diabetes Metab Res Rev* 2001; 17: 374-379.
37. El-Serag HB, Tran T, Everhart JE. Diabetes increases the risk of chronic liver disease and hepatocellular carcinoma. *Gastroenterology* 2004; 126: 460-468.
38. Yu MC, Tong MJ, Govindarajan S, Henderson BE. Nonviral risk factors for hepatocellular carcinoma in a low-risk population, the non-Asians of Los Angeles County, California. *J Natl Cancer Inst* 1991; 83: 1820-1826.
39. Lonardo A, Ballestri S, Targher G, Loria P. Diagnosis and management of cardiovascular risk in nonalcoholic fatty liver disease. Expert Rev Gastroenterol Hepatol 2015; 9: 629-650.
40. Lonardo A, Loria P. Potential for statins in the chemoprevention and management of hepatocellular carcinoma. J Gastroenterol Hepatol 2012; 27: 1654-1664.
41. Magee C. Kidney disease and death from cancer. *Am J Kidney Dis* 2014; 63: 7-9.
42. Izzedine H, Perazella MA. Onco-nephrology: an appraisal of the cancer and chronic kidney disease links. *Nephrol Dial Transplant* 2015; 30: 1979-1988.
43. Non-alcoholic Fatty Liver Disease Study G, Lonardo A, Bellentani S et al. Epidemiological modifiers of non-alcoholic fatty liver disease: Focus on high-risk groups. Dig Liver Dis 2015; 47: 997-1006.

For peer review only

For peer review only - http://bmjopen.bmj.com/site/about/guidelines.xhtml

44. Rinaldi L, Nascimbeni F, Giordano M et al. Clinical features and natural history of cryptogenic cirrhosis compared to hepatitis C virus-related cirrhosis. World J Gastroenterol 2017; 23: 1458-1468.

45. Piscaglia F, Svegliati-Baroni G, Barchetti A et al. Clinical patterns of hepatocellular carcinoma in nonalcoholic fatty liver disease: A multicenter prospective study. Hepatology 2016; 63: 827-838.

46. Chen CH, Dai CY, Hou NJ, et al. Etiology, severity and recurrence of acute pancreatitis in southern taiwan. *J Formos Med Assoc* 2006; 105: 550-555.

47. Bruix J, Sherman M, Practice Guidelines Committee AAftSoLD. Management of hepatocellular carcinoma. *Hepatology* 2005; 42: 1208-1236.

48. Makuuchi M, Kokudo N, Arii S, et al. Development of evidence-based clinical guidelines for the diagnosis and treatment of hepatocellular carcinoma in Japan. *Hepatol Res* 2008; 38: 37-51.

49. Llovet JM, Di Bisceglie AM, Bruix J, et al. Design and endpoints of clinical trials in hepatocellular carcinoma. *J Natl Cancer Inst* 2008; 100: 698-711.

50. Takayama T, Makuuchi M, Kojiro M, et al. Early hepatocellular carcinoma: pathology, imaging, and therapy. *Ann Surg Oncol* 2008; 15: 972-978.

51. Ye SL, Takayama T, Geschwind J, et al. Current approaches to the treatment of early hepatocellular carcinoma. *Oncologist* 2010; 15 Suppl 4: 34-41.

52. Kuo MJ, Chen HH, Chen CL, et al. Cost-effectiveness analysis of population-based screening of hepatocellular carcinoma: Comparing ultrasonography with two-stage screening. *World J Gastroenterol* 2016; 22: 3460-3470.

53. Bertakis KD, Azari R, Helms LJ, et al. Gender differences in the utilization of health care services. *J Fam Pract* 2000; 49: 147-152.

For peer review only - http://bmjopen.bmj.com/site/about/guidelines.xhtml

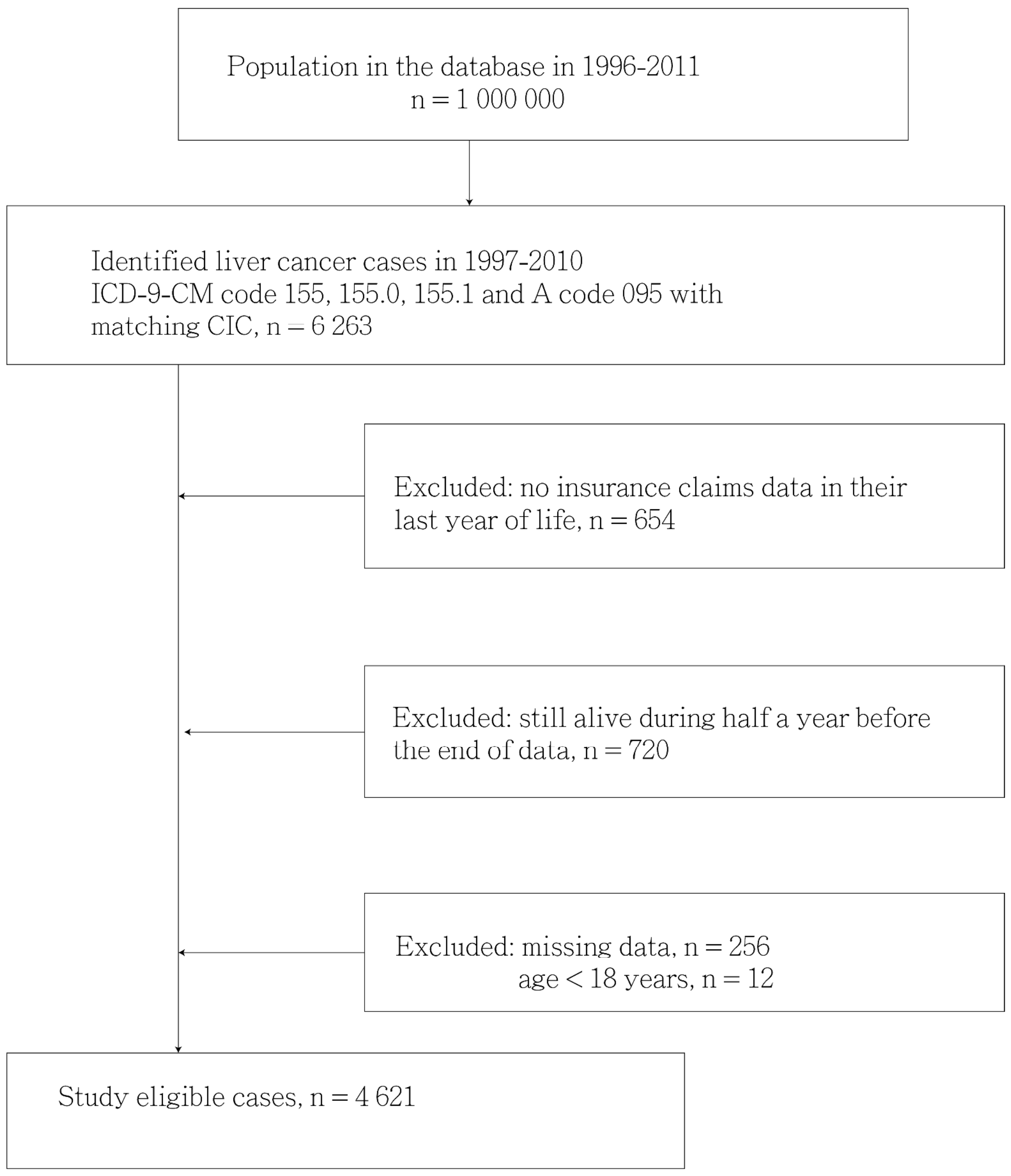

467x543mm (300 x 300 DPI)

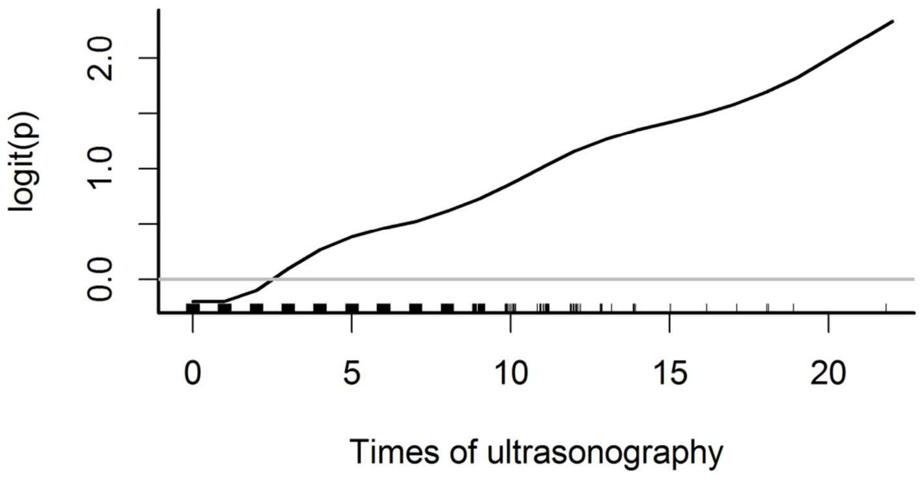

101x67mm (300 x 300 DPI)

view only

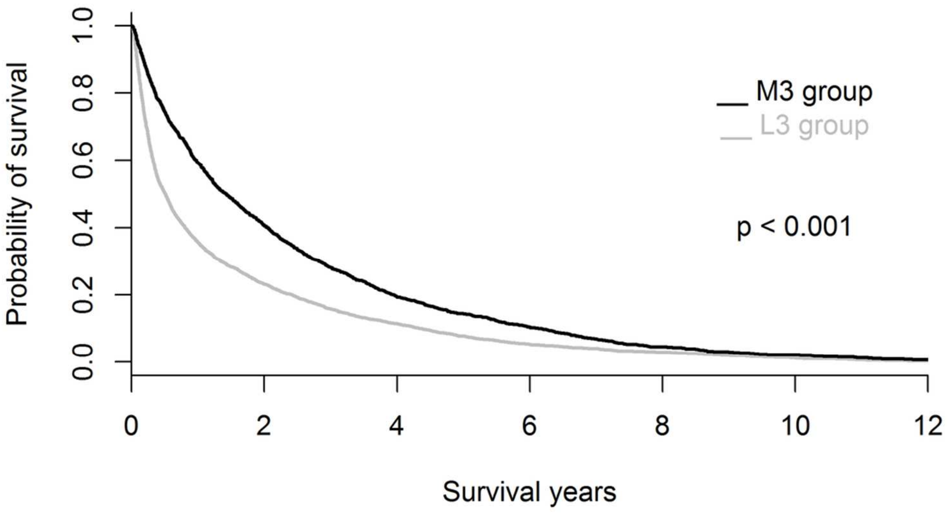

101x67mm (300 x 300 DPI)

view only

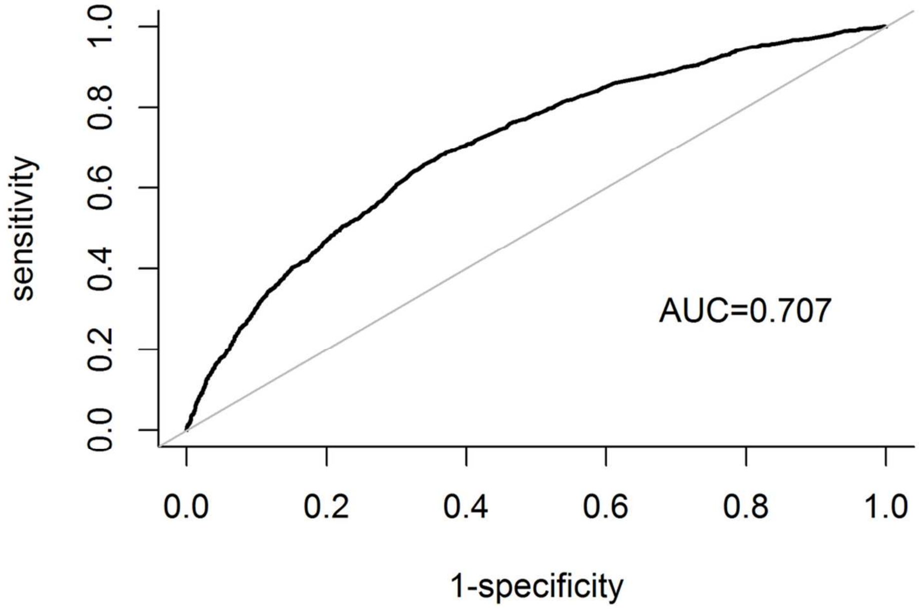

101x81mm (300 x 300 DPI)

For peer review only

STROBE Statement—Checklist of items that should be included in reports of ***cohort studies***

	Item No	Recommendation
**Title and abstract**	1	(*a*) Indicate the study's design with a commonly used term in the title or the abstract (page 2)
		(*b*) Provide in the abstract an informative and balanced summary of what was done and what was found (page 2)
**Introduction**		
Background/rationale	2	Explain the scientific background and rationale for the investigation being reported (page 5-6)
Objectives	3	State specific objectives, including any prespecified hypotheses (page 6)
**Methods**		
Study design	4	Present key elements of study design early in the paper (page 6)
Setting	5	Describe the setting, locations, and relevant dates, including periods of recruitment, exposure, follow-up, and data collection (page 6)
Participants	6	(*a*) Give the eligibility criteria, and the sources and methods of selection of participants. Describe methods of follow-up (page 7)
		(*b*) For matched studies, give matching criteria and number of exposed and unexposed (not indicated)
Variables	7	Clearly define all outcomes, exposures, predictors, potential confounders, and effect modifiers. Give diagnostic criteria, if applicable (page 7-8)
Data sources/ measurement	8*	For each variable of interest, give sources of data and details of methods of assessment (measurement). Describe comparability of assessment methods if there is more than one group (page 7-9)
Bias	9	Describe any efforts to address potential sources of bias (page 7)
Study size	10	Explain how the study size was arrived at (page 7)
Quantitative variables	11	Explain how quantitative variables were handled in the analyses. If applicable, describe which groupings were chosen and why (page 8)
Statistical methods	12	(*a*) Describe all statistical methods, including those used to control for confounding (page 9)
		(*b*) Describe any methods used to examine subgroups and interactions (page 9)
		(*c*) Explain how missing data were addressed (Page 7, figure 1)
		(*d*) If applicable, explain how loss to follow-up was addressed (page 7, figure 1)
		(*e*) Describe any sensitivity analyses (page 10)
**Results**		
Participants	13*	(a) Report numbers of individuals at each stage of study—eg numbers potentially eligible, examined for eligibility, confirmed eligible, included in the study, completing follow-up, and analysed (page 10)
		(b) Give reasons for non-participation at each stage (page 10, figure 1)
		(c) Consider use of a flow diagram (page 10, figure 1)
Descriptive data	14*	(a) Give characteristics of study participants (eg demographic, clinical, social) and information on exposures and potential confounders (page 11)
		(b) Indicate number of participants with missing data for each variable of interest (page 10, figure 1)
		(c) Summarise follow-up time (eg, average and total amount) (page 10)
Outcome data	15*	Report numbers of outcome events or summary measures over time (page 11)
Main results	16	(*a*) Give unadjusted estimates and, if applicable, confounder-adjusted estimates and their precision (eg, 95% confidence interval). Make clear which confounders were

For peer review only - http://bmjopen.bmj.com/site/about/guidelines.xhtml

		adjusted for and why they were included (page 11)
		(*b*) Report category boundaries when continuous variables were categorized (page 11)
		(*c*) If relevant, consider translating estimates of relative risk into absolute risk for a meaningful time period (page 13)
Other analyses	17	Report other analyses done—eg analyses of subgroups and interactions, and sensitivity analyses (page 13)
**Discussion**		
Key results	18	Summarise key results with reference to study objectives (page 13)
Limitations	19	Discuss limitations of the study, taking into account sources of potential bias or imprecision. Discuss both direction and magnitude of any potential bias (page 17)
Interpretation	20	Give a cautious overall interpretation of results considering objectives, limitations, multiplicity of analyses, results from similar studies, and other relevant evidence (page 13-14)
Generalisability	21	Discuss the generalisability (external validity) of the study results (page 14)
**Other information**		
Funding	22	Give the source of funding and the role of the funders for the present study and, if applicable, for the original study on which the present article is based (page 18)

*Give information separately for exposed and unexposed groups.

Note: An Explanation and Elaboration article discusses each checklist item and gives methodological background and published examples of transparent reporting. The STROBE checklist is best used in conjunction with this article (freely available on the Web sites of PLoS Medicine at http://www.plosmedicine.org/, Annals of Internal Medicine at http://www.annals.org/, and Epidemiology at http://www.epidem.com/). Information on the STROBE Initiative is available at http://www.strobe-statement.org.

For peer review only

For peer review only - http://bmjopen.bmj.com/site/about/guidelines.xhtml

BMJ Open

Effect of Ultrasonography Surveillance in Patients with Liver Cancer: A Population-Based Longitudinal Study

Journal:	*BMJ Open*
Manuscript ID	bmjopen-2017-015936.R2
Article Type:	Research
Date Submitted by the Author:	18-May-2017
Complete List of Authors:	Chiang, Jui-Kun<br>Lin , Chih-Wen<br>Kao, Yee-Hsin; Family Medicine; Tainan Municipal Hospital
**Primary Subject Heading**:	General practice / Family practice
Secondary Subject Heading:	Gastroenterology and hepatology, Epidemiology
Keywords:	PREVENTIVE MEDICINE, Hepatobiliary disease < GASTROENTEROLOGY, Hepatobiliary tumours < ONCOLOGY, PRIMARY CARE

SCHOLARONE™ Manuscripts

Effect of Ultrasonography Surveillance in Patients with Liver Cancer: A Population-Based Longitudinal Study

Jui-Kun Chiang[1], M.D., MSc, Lin Chih-Wen[2, 3] M.D., MBA, Yee-Hsin Kao[4], M.D.

[1]Department of Family Medicine, Dalin Tzu Chi Hospital, Buddhist Tzu Chi Medical Foundation, Dalin, Chiayi, Taiwan

[2]Department of Medical Imaging, Dalin Tzu Chi Hospital, Buddhist Tzu Chi Medical Foundation, Dalin, Chiayi, Taiwan.

[3]School of Medicine, Tzu Chi University, Hualien, Taiwan.

[4]Department of Family Medicine, Tainan Municipal Hospital, Tainan, Taiwan.

Running head: Ultrasonography surveillance on survival of liver cancer patients

Corresponding author: Yee-Hsin Kao:
Department of Family Medicine
Tainan Municipal Hospital
670 Chung-Te Road
Tainan 701, Taiwan
E-mail: m2200767@gmail.com
Tel: +886 6 260-9926 × 22226
Fax: +886 6 260-9900

E-mail for Jui-Kun Chiang: jkch68@gmail.com
E-mail for Lin Chih-Wen: dalinchiayi@gmail.com

Number of manuscript Pages:
Number of Figures: 4
Number of Tables: 5
Words counts: Abstract: 255, Text: 3381 (introduction: 502, discussion: 1192)
Number of References: 53

For peer review only

For peer review only - http://bmjopen.bmj.com/site/about/guidelines.xhtml

Abstract

Objective Liver cancer is a growing global public health problem. Ultrasonography is an imaging tool widely used for the early diagnosis of liver cancer. However, the effect of ultrasonography surveillance (US) on the survival of patients with liver cancer is unknown. Therefore, this study examined the association between survival and US frequency during the 2 years preceding patients' liver cancer diagnosis.

Methods This population-based longitudinal study was conducted in Taiwan, a region with high liver cancer incidence, by using the National Health Insurance Research Database. We compared survival between patients who received US three times or more (≥3 group) and less than three times (<3 group) during the 2 years preceding their liver cancer diagnosis, and identified the predictors for the ≥3 group.

Results This study enrolled 4621 patients with liver cancer who had died between 1997 and 2010. The median survival rate was higher in the ≥3 group (1.42 years) than in the <3 group (0.51 years). Five-year survival probability was also significantly higher in the ≥3 group (14.4%) than in the <3 group (7.7%). The multivariate logistic regression results showed that the three most common positive predictors for receiving three or more US sessions were indications of viral hepatitis, gallbladder diseases, and kidney–urinary–bladder diseases; the most common negative predictors for receiving three or more US sessions were male sex and indications of abdominal pain.

For peer review only - http://bmjopen.bmj.com/site/about/guidelines.xhtml

Conclusion Patients with liver cancer who received US three times or more during the 2 years preceding their liver cancer diagnosis exhibited a higher 5-year survival probability.

Keywords: ultrasonography surveillance (US), liver cancer, survival

Strengths and limitations of this study

This is a nationwide, population-based study that was conducted in Taiwan, a region that has a high incidence of liver cancer.

We determined that patients' 5-year survival probability was significantly higher among those who had received three or more sessions of US (14.4%) than among those who received less than three sessions of US (7.7%).

The survival results associated with patients receiving three or more sessions of US 2 years prior to their liver cancer diagnosis may be applicable to the general population in Taiwan because of the high incidence of liver cancer.

No information on cancer stage was available in the dataset, and some crucial potentially confounding variables were also not available for performing statistical adjustment.

The cause of death was not recorded in the insurance claims data, and therefore we cannot establish associations between liver cancer and mortality.

For peer review only - http://bmjopen.bmj.com/site/about/guidelines.xhtml

Introduction

Liver cancer is a growing global public health problem and is the fifth and ninth most common cancer in men and women worldwide, respectively. Because of its poor outcome, it is also the second most common cause of cancer-related mortality, accounting for nearly 746,000 deaths worldwide in 2012.[1] Eastern and Southeastern Asia, including Taiwan, are regions with a particularly high incidence of liver cancer. In Taiwan, liver cancer has been the second leading cause of cancer-related mortality since 2004, and it accounted for 8,116 (18.6%) of the 43,665 cancer deaths in 2012.[2] The known risk factors for liver cancer are viral infection (hepatitis B virus [HBV] and hepatitis C virus [HCV] infections), alcohol consumption and Alfa toxins, diabetes, and metabolic (e.g., nonalcoholic fatty liver disease [NAFLD] and hereditary hemochromatosis) and immune-related (e.g., primary biliary cirrhosis and autoimmune hepatitis) diseases.[3]

One previous study reported that chronic HBV and HCV infections are the primary risk factors for liver cancer.[4] To improve the effective control and treatment of viral hepatitis, two programs have been implemented in Taiwan. The first is the mass vaccination program targeted against HBV, which has considerably reduced the HBV carrier rate among children and adolescents and consequently reduced the incidence of childhood liver cancer in Taiwan.[5-8] The second program is a pilot

For peer review only - http://bmjopen.bmj.com/site/about/guidelines.xhtml

program initiated in October 2003 by the Taiwanese Center for Disease Control, entitled “Strengthening of treatment for chronic hepatitis B and C under the National Health Insurance.” This program has attempted to ensure that patients with chronic hepatitis B and C are registered, so that they can benefit from appropriate diagnosis, monitoring, and treatment.[9] Accordingly, the incidence of liver cancer significantly decreased by an average annual percentage of 1.1% from 2002 to 2012; during this same period, the 5-year relative survival rates for liver cancer were 52.0% for stage I, 2.9% for stage IV, and 28.9% for all stages.[10]

A diagnosis of hepatocellular cancer is based on a combination of radiological, serological, and histopathological criteria. Some studies have reported that alpha-fetoprotein determination lacks adequate sensitivity and specificity for effective surveillance;[11 12] therefore, surveillance must be based on ultrasonography, which has a recommended screening interval of 6 months.[13 14] A previous study reported that ultrasonography has a sensitivity of approximately 63% and a high specificity of approximately 85%–90% for the detection of early-stage liver cancer.[12] Moreover, ultrasonography is a noninvasive and safe method, is well accepted by patients, and is relatively inexpensive.

Research has indicated that most patients with hepatocellular carcinoma have a low rate of survival. However, patients with early-stage hepatocellular carcinoma who

For peer review only - http://bmjopen.bmj.com/site/about/guidelines.xhtml

receive potentially curative therapy exhibit considerably improved survival (5-year survival = 40%–70%).[15] One systemic review provided very-low-strength evidence about the effects of hepatocellular carcinoma screening on mortality in patients with chronic hepatitis.[16] Nevertheless, regular surveillance with abdominal ultrasonography might facilitate the early detection of liver cancer, thus improving the survival of at-risk patients. This study therefore examined the effect of ultrasonography surveillance (US) on survival. Specifically, the association between patient survival and the frequency of US during the 2 years preceding liver cancer diagnosis was determined, and the predictors of receiving more US sessions were identified.

Methods

Data source and patient identification

For this nationwide population-based study, we analyzed the data from the National Health Insurance Research Database (NHIRD) of Taiwan. The National Health Insurance (NHI) program, established in 1995, is a single-payer health insurance system that covers 99.9% of all residents in Taiwan. Moreover, 97% of medical providers nationwide are affiliated with the program.[17] The Longitudinal Health Insurance Database (LHID) is a nationwide representative database containing

For peer review only - http://bmjopen.bmj.com/site/about/guidelines.xhtml

all of the original claims data for the period of 1996–2011 for one million NHI beneficiaries randomly sampled from the 23.22 million NHI enrollees. The availability of this population-based database has stimulated and facilitated academic research in various scientific disciplines, particularly in the field of health research.[18] In addition, the accuracy of diagnoses registered in the NHIRD has been validated using five-digit International Classification of Diseases, Ninth Revision, Clinical Modification (ICD-9-CM) codes, including those for diabetes and cancer, which confirms the reliability of the data source.[19] In Taiwan, all cancer patients are designated as having a catastrophic illness. For this study, we identified patients with liver cancer and catastrophic illness designations in the LHID and followed up with these patients until December 2011. ICD-9-CM and A codes were used to define liver cancer (namely, 155, 155.0, 155.1, and A095).

Study sample

This study included patients aged ≥18 years who had received a first-time diagnosis of liver cancer between January 1, 1997 and December 31, 2010. The NHIRD was used to identify the patients, and their diagnoses of cancer were confirmed using the Registry of Catastrophic Illness. Patients were excluded if they had made no insurance claims during their final year of life, were still alive during the 6 months before the end of the dataset, or had missing data. A total of 4621 patients

For peer review only - http://bmjopen.bmj.com/site/about/guidelines.xhtml

with liver cancer were enrolled in this study (Figure 1).

Frequency of abdominal ultrasonography surveillance during the 2 years preceding liver cancer diagnosis

We classified the study population into two groups based on their frequency of receiving US during the 2 years preceding their liver cancer diagnosis: the <3 group (patients who had received US less than three times; n = 3149) and the ≥3 group (patients who had received US three times or more; n = 1472).

We classified the diseases that are indicators for ultrasonography into seven groups using their respective ICD-9-CM diagnosis codes. The seven disease groups are as follows: (1) liver-related diseases, including chronic hepatitis, cirrhosis, and other liver disorders (ICD-9 codes: 571, 571.x, 572, 572.0, 573, and 573.x); (2) viral hepatitis infections (ICD-9 codes for HBV: V0261, 070.20–070.23, and 070.30–070.33; for HCV: V0262, 070.41, 070.44, 070.51, and 070.54); (3) gallbladder and biliary tract-related diseases (ICD-9 codes: 574–576); (4) abdominal pain conditions (ICD-9 codes: 789.0x, 789.4x, 789.6x, and 789.9); (5) kidney–urinary–bladder (KUB) diseases (ICD-9 codes: 591, 592, 592.0, 592.1, 592.9, 593, and 593.2); (6) pancreatic diseases (ICD-9 code: 577.x); and (7) other conditions, such as hepatomegaly, splenomegaly, abdominal or pelvic mass, and ascites (ICD-9 codes: 7891, 7892, 7893x, and 7895).

For peer review only - http://bmjopen.bmj.com/site/about/guidelines.xhtml

Definition of variables

Charlson comorbidity index (CCI) scores were calculated by examining patients' ICD-9-CM diagnoses and procedure codes recorded in the year prior to their liver cancer diagnosis, according to the Deyo method. After referring to Klabundle *et al.*, we calculated CCI scores for both inpatient and outpatient claims.[20-22]

The study protocol was reviewed and approved by the Research Ethics Committee of Dalin Tzu Chi Hospital, Buddhist Tzu Chi Medical Foundation, Taiwan (Approval No. B10304018). Because the NHIRD contains only deidentified secondary data, the review board waived the requirement for informed consent.

Statistical analyses

In this paper, continuous variables are expressed in mean ± standard deviation, and categorical variables are presented in frequency and percentage. For the univariate analysis, a two-sample *t* test, Wilcoxon rank-sum test, chi-square test, and Fisher's exact test were performed to examine differences in the distribution of the continuous and categorical variables between the two groups. The survival duration (years) was defined as the duration from the day of diagnosis to the day of death. In addition, generalized additive models (GAMs) were fitted to detect the potential nonlinear effects of continuous covariates, and to identify the appropriate cutoff points for discretizing continuous covariates (if necessary) during the stepwise

For peer review only

For peer review only - http://bmjopen.bmj.com/site/about/guidelines.xhtml

variable selection. Computationally, the VGAM function (with the default values of smoothing parameters) of the VGAM package was used to fit GAMs for the binary outcome in R.

Cox proportional hazard regression models were used to estimate the event-specific hazard ratios (HRs) and 95% confidence intervals (CIs) of all the variables listed in Tables 1 and 2. Subsequently, a multivariate analysis was conducted by fitting the multiple logistic regression models (through a stepwise variable selection procedure) to identify the crucial predictors of receiving US three times or more during the 2 years preceding a liver cancer diagnosis. The goodness-of-fit (GOF) of the final logistic regression model was assessed by estimating the area under the curve (AUC; also called the c statistic), where $0 \leq c \leq 1$, and by conducting the Hosmer–Lemeshow GOF test. In practice, $c \geq 0.7$ is an acceptable level of discrimination power for a fitted logistic regression model and $p > 0.05$ in the Hosmer–Lemeshow test indicates a good fit for the logistic regression model. Moreover, a variance inflation factor of ≥10 for continuous covariates or ≥2.5 for categorical covariates indicates that multicollinearity occurs among some of the covariates in a fitted logistic regression model. All statistical analyses were performed using the R 3.0.2 software (R Foundation for Statistical Computing, Vienna, Austria). Statistical significance was set at $p \leq 0.05$.

For peer review only - http://bmjopen.bmj.com/site/about/guidelines.xhtml

Results

We enrolled 4621 adult patients (3287 men and 1334 women; ratio = 2.46:1) with liver cancer who died during 1997–2010. Figure 1 depicts the study design. Specifically, we explored the association between the frequency of US during the 2 years preceding a patient's liver cancer diagnosis and that patient's 5-year survival probability. The optimal cutoff point for receiving US to ensure a higher 5-year survival probability was set at three sessions (Figure 2).

Table 1 summarizes the demographic characteristics of the study patients, and indicates that those in the ≥3 group were more likely to be female ($p < 0.001$) and were more likely to have the comorbidities of viral hepatitis (HBV or HCV infection) ($p < 0.001$), cirrhosis ($p < 0.001$), diabetes ($p < 0.001$), hypertension ($p < 0.001$), or stroke ($p = 0.001$). Notably, the median survival of the ≥3 group (1.42 years) was higher than that of the <3 group (0.51 years) ($p < 0.001$). The indicators for abdominal US used in this study are summarized in Table 2. The most frequent indicators were viral hepatitis (2580, 55.8%), gallbladder diseases (1690, 36.6%), and abdominal pain (1387, 30.0%). In this study, all indicators, except for abdominal pain ($p = 0.098$), were significantly higher in the ≥3 group than in the <3 group.

Compared with the <3 group, significantly higher proportions of the ≥3 group

For peer review only - http://bmjopen.bmj.com/site/about/guidelines.xhtml

received all possible treatments for liver cancer, except for radiotherapy (p = 0.097); the ≥3 group also had significantly higher 5-year survival probability than did the <3 group (14.4% vs. 7.7%, respectively; $p < 0.001$) (Table 3). From the multivariate Cox proportional hazard regression model, the positive significant predictors of death were male sex (HR: 1.14, 95% CI: 1.07–1.22), older age (HR: 1.02, 95% CI: 1.01–1.03), diabetes (HR: 1.13, 95% CI: 1.04–1.22), and a high CCI score (HR: 1.07, 95% CI: 1.05–1.08). Conversely, the negative significant predictors of death were hypertension (HR: 0.83, 95% CI: 0.78–0.89), stroke (HR: 0.78, 95% CI: 0.72–0.84), chronic kidney diseases (CKDs) (HR: 0.84, 95% CI: 0.74–0.92), viral hepatitis (HBV or HCV infection) (HR: 0.79, 95% CI: 0.72–0.88), and receiving US three times or more during the 2 years preceding liver cancer diagnosis (HR: 0.80, 95% CI: 0.75-0.85).

The significant indicators for US among the patients in this study included KUB diseases (HR: 0.92, 95% CI: 0.86–0.995), gallbladder diseases (HR: 0.90, 95% CI: 0.85–0.96), chronic hepatitis (except for HBV or HCV infection) (HR: 0.90, 95% CI: 0.81–0.999), and pancreatic diseases (HR: 0.80, 95% CI: 0.71–0.91). Moreover, the primary complications of liver cancer were upper gastrointestinal bleeding (gastric ulcer or duodenal ulcer) (HR: 0.92, 95% CI: 0.86–0.98) and esophageal variceal bleeding (HR: 0.90, 95% CI: 0.83–0.97), and the main treatments that the patients received for liver cancer were transcatheter arterial chemoembolization (TACE) (HR:

For peer review only - http://bmjopen.bmj.com/site/about/guidelines.xhtml

0.82, 95% CI: 0.76–0.89), radiotherapy (HR: 0.81, 95% CI: 0.75–0.87), radiofrequency ablation (RFA) (HR: 0.79, 95% CI: 0.68–0.93), chemotherapy (HR: 0.71, 95% CI: 0.67–0.76), resection (HR: 0.67, 95% CI: 0.61–0.73), and percutaneous ethanol injection (PEI) (HR: 0.64, 95% CI: 0.57–0.70). Furthermore, the demographic factors significantly linked with greater US frequency were employment (HR: 0.89, 95% CI: 0.83–0.95) and living in Northern Taiwan (HR: 0.91, 95% CI: 0.85–0.97) (Table 4).

Survival probability was significantly higher in the ≥3 group than in the <3 group ($p < 0.001$; Figure 3). According to the multivariate logistic regression model, the significant positive predictors for survival in the ≥3 group were viral hepatitis (HBV or HCV infection) (odds ratio [OR]: 3.66, 95% CI: 2.76–4.85), gallbladder diseases (OR: 1.82, 95% CI: 1.59–2.09), KUB diseases (OR: 1.80, 95% CI: 1.53–2.11), pancreatic diseases (OR: 1.79, 95% CI: 1.37–2.34), chronic hepatitis (except for viral hepatitis; OR: 1.75, 95% CI: 1.31–2.34), CKDs (OR: 1.60, 95% CI: 1.30–1.96), diabetes (OR: 1.56, 95% CI: 1.31–1.86), US indications of other conditions (OR: 1.47, 95% CI: 1.29-1.69), hypertension (OR: 1.17, 95% CI: 1.01–1.34), and living in Central Taiwan (OR: 1.16, 95% CI: 1.01–1.34). Conversely, the significant negative predictors for survival in the ≥3 group were male sex (OR: 0.60, 95% CI: 0.52–0.69) and the US indication of abdominal pain (OR: 0.81, 95% CI:

For peer review only - http://bmjopen.bmj.com/site/about/guidelines.xhtml

0.70–0.93) (Table 5).

The results of the Hosmer–Lemeshow test indicated a good fit (p = 0.156) and that the AUC was acceptable (0.707, 95% CI: 0691–0723) (Figure 4).

Discussion

In this study, we found that patients with liver cancer who had received US three times or more during the 2 years preceding their liver cancer diagnosis exhibited high 5-year survival compared with those who had received US less than three times during that same period (14.4% vs. 7.7%). We also determined that, compared with the <3 group, higher proportions of the ≥3 group received treatment for early-stage liver cancer (e.g., PEI, hepatectomy, RFA, or liver transplantation). As illustrated in Figure 2, there was a linear dose-response relationship between US frequency and the logit of 5-year survival probability; a similar result was also reported in a previous study.[23] Further cost-effectiveness analyses are needed to justify recommending three or more sessions of US to people at a high risk of liver cancer diagnosis or to the general population in an area with a high incidence of liver cancer.

According to one previous trial, biannual US for patients with HBV infection reduces hepatocellular carcinoma mortality by 37%.[24] Another cohort study reported that patients with viral hepatitis who receive routine US exhibit higher 5-year survival than do those who did not receive routine US (31.84% vs. 20.67%, respectively).[25]

For peer review only - http://bmjopen.bmj.com/site/about/guidelines.xhtml

Other scholars have indicated that certain populations at a high risk for developing hepatocellular carcinoma benefit from more intensive US.[26-30] One trial study revealed that early-stage hepatocellular carcinoma was more likely to be detected in patients with viral hepatitis who received US at 4-month intervals than in those who received US at 12-month intervals; however, patients' overall survival was not different at the 4-year follow-up.[31] In another systemic review, the effects of hepatocellular carcinoma screening on mortality in patients with chronic hepatitis were found to be uncertain.[16]

In short, receiving US at a high frequency has no confirmed beneficial effect on the 5-year survival probability of patients with hepatocellular carcinoma. This finding may be because the early detection of liver cancer improves the rate of curative treatments. However, the prognosis of patients with liver cancer is not solely related to the tumor stage, [32] and regular follow-up for possible recurrence and treatment is vital. Although the survival of patients with liver cancer has improved overall in the past decade, physicians caring for at-risk patients should provide regular high-quality screening, including ultrasonography.[13]

Although advanced diagnostic techniques, such as dynamic multiphasic multidetector-row computed tomography and magnetic resonance imaging, are the standard diagnostic methods for the noninvasive diagnosis of liver cancer,

For peer review only - http://bmjopen.bmj.com/site/about/guidelines.xhtml

ultrasonography still plays a crucial role in liver cancer surveillance.[33] In Taiwan, the recommended intervals for US screening is 6 months for patients with viral hepatitis infection and 3 months for patients with cirrhosis. Ultrasonography can be performed in hospital and clinics, if indicated, and the cost of each ultrasonography screening (USD 23.1) is covered by the NHI program.

In the present study, we found that patients with chronic hepatitis (both HBV and HCV, and non-HBV and -HCV infections), diabetes, CKDs, cerebral vascular accident (CVA), hypertension, gallbladder diseases, KUB diseases, pancreatic diseases, and other US-indicating conditions (i.e., hepatomegaly, splenomegaly, abdominal or pelvic mass, and ascites) were more likely to receive US three times or more during the 2 years preceding their liver cancer diagnosis. One reason that patients with diabetes in this study were more likely to receive US three times or more might be because these patients are at a high risk of primary liver cancer.[34-36] Notably, the association between diabetes and liver cancer is significantly strengthened when the history of diabetes is longer than 10 years.[37 38] Some studies have also suggested that insulin might enhance the risk of hepatocellular cancer, whereas certain oral glucose-lowering medications and statins may decrease the risk of hepatocellular cancer.[39 40]

Similarly, patients with CKD in this study were more likely to receive US three

For peer review only - http://bmjopen.bmj.com/site/about/guidelines.xhtml

times or more, most likely because of the close link between kidney diseases and cancer.[41] Indeed, cancer has become increasingly recognized as a complication and a major cause of morbidity and mortality in the CKD population.[42] Finally, the reason that patients with CVA, hypertension, gallbladder diseases, KUB diseases, pancreatic diseases, or other US-indicating conditions in this study were more likely to receive US three times or more is probably because patients with more comorbidities and physician visits are more likely to undergo more laboratory tests and imaging studies, including US, if they experience any discomfort.

The chances of developing NAFLD are enhanced by type 2 diabetes, arterial hypertension, obesity, metabolic syndrome, mixed hyperlipidemia, and familial hypobetalipoproteinemia, all of which are the chief metabolic modifiers of NAFLD risk.[43-45] In this study, NAFLD was included in the group of liver-related diseases alongside chronic hepatitis, cirrhosis, and other liver disorders. However, the imprecise classification for NAFLD is one of the claims-data-related limitations of this study. Instead, we classified the diseases that are the indicators for US into seven groups; patients with these indicators easily received US due to its widespread availability in Taiwan. Another limitation of this study is that baseline data were not recorded in the claims data, and thus could not be investigated. For example, the underlying causes of pancreatic disease include alcohol abuse, biliary disease,

For peer review only - http://bmjopen.bmj.com/site/about/guidelines.xhtml

hyperlipidemia, and other risk factors, etiologies or idiopathic diseases;[46] however, it is unknown whether patients in the present study who had pancreatic disease were also diagnosed with any of these baseline factors or diseases.

As noted earlier, higher proportions of the ≥3 group received treatment for early-stage liver cancer, such as PEI, hepatectomy, RFA, or liver transplantation, than did the <3 group. Such treatments have similarly been reported as therapies for early-stage hepatocellular carcinoma in previous studies.[47-50] We also determined that higher proportions of the ≥3 group received TACE compared with the <3 group, although the therapeutic effects of TACE for early-stage hepatocellular carcinoma are unclear.[51]

One previous study reported that mass screening for liver cancer by using US in high-endemic regions, including Taiwan, is cost-effective and recommended.[52] In this study, the significant positive predictors for survival in the ≥3 group were viral hepatitis, gallbladder diseases, KUB diseases, pancreatic diseases, chronic hepatitis (except viral infections), diabetes, US indications of other conditions, hypertension, and living in Central Taiwan. By contrast, the significant negative predictors for survival in the ≥3 group were male sex and the US indication of abdominal pain. The reason that these factors are negative predictors might be because men are less likely to seek medical attention than are women,[53] and because after seeking medical

For peer review only - http://bmjopen.bmj.com/site/about/guidelines.xhtml

attention for one abdominal pain event, patients do not return for subsequent follow-up, respectively. However, we could not confirm the precise reason that these factors are negative predictors because such information is not available from the claims data; this is another limitation of this study. Furthermore, although the patients with these negative predictors were more likely to die of liver cancer, official cause of death is not recorded in the claims data, and such causal links could not be confirmed in this study.

Finally, this was an observational cohort study, and its validity may be jeopardized by confounding variables, such as cancer stage, body mass index, smoking, and alcohol consumption, that were not included in the dataset and thus were not measured. We therefore suggest that randomized clinical trials be conducted before the conclusions of the present study are applied to clinical practice in Taiwan and elsewhere.

Conclusions

The 5-year survival probability of patients with liver cancer may improve if they receive abdominal US three times or more during the 2 years preceding their liver cancer diagnosis. Additional studies should prospectively investigate whether abdominal US can be used to identify early-stage liver cancer and examine whether further treatment with regular follow-up improves survival.

For peer review only - http://bmjopen.bmj.com/site/about/guidelines.xhtml

Acknowledgments

This study is based in part on data from the National Health Insurance Research Database, provided by the Bureau of National Health Insurance of the Department of Health and managed by the National Health Research Institutes. The interpretations and conclusions contained herein do not represent those of the Bureau of National Health Insurance, Department of Health, or National Health Research Institutes.

Footnotes

Contributors: JKC, LCW, and YHK designed, conducted, and drafted the manuscript. JKC analyzed the data. All authors contributed to the manuscript, critically revised drafts for intellectual content, and read and approved the final manuscript.

Funding: JK Chiang received research grants from Dalin Tzu Chi Hospital, Buddhist Tzu Chi Medical Foundation (DTCRD105 (2)-E-22). The funding source had no involvement in the study design; collection, analysis, and interpretation of data; writing of the report; or decision to submit the article for publication.

Competing interests: None declared.

Ethics approval: The study protocol was reviewed and approved by the Research Ethics Committee of Dalin Tzu Chi Hospital, Buddhist Tzu Chi Medical Foundation,

For peer review only - http://bmjopen.bmj.com/site/about/guidelines.xhtml

Taiwan (Approval No. B10304018).

Data sharing statement: No additional data are available.

For peer review only - http://bmjopen.bmj.com/site/about/guidelines.xhtml

Table 1. Comparison of demographic characteristics between the <3 and ≥3 groups

Variable	<3 group, n (%)	≥3 group, n (%)	*p* value
Total number (%)	3149 (68.1)	1472 (31.9)	
Age (years)	63.04 ± 13.92	63.77 ± 11.76	0.474
Gender			< 0.001
female	807 (25.6)	527 (35.8)	
male	2342 (74.4)	945 (64.2)	
Survival*	0.51 (0.17, 1.81)	1.42 (0.48, 3.34)	< 0.001
HBV	1119 (35.5)	619 (42.1)	< 0.001
HCV	660 (21.0)	620 (42.1)	< 0.001
Diabetes	535 (17.0)	361 (24.5)	< 0.001
CKD	276 (8.8)	232 (15.8)	< 0.001
CVA	535 (17.0)	297 (20.2)	0.001
Hypertension	1173 (37.2)	691 (46.9)	< 0.001
Cirrhosis	2048 (65.0)	1183 (80.4)	< 0.001
Employment (yes)	1873 (59.5)	891 (60.5)	0.499
Northern area in Taiwan	1063 (33.8)	467 (31.7)	0.178
Central area in Taiwan	888 (28.2)	461 (31.3)	0.031
Southern area in Taiwan	1032 (32.8)	480 (32.6)	0.920
Eastern area in Taiwan	153 (4.9)	57 (3.9)	0.150

Abbreviations: HBV, hepatitis B virus; HCV, hepatitis C virus; CKD, chronic kidney disease; CVA, cerebral vascular accident; CCI, Charlson comorbidity index

Survival*: median (first quartile, third quartile)

For peer review only - http://bmjopen.bmj.com/site/about/guidelines.xhtml

Table 2. Ultrasonography surveillance indicators in the <3 and ≥3 groups

Variable	Total number (%)	<3 group, n (%)	≥3 group, n (%)	*p* value
Viral hepatitis (HBV, HCV)	2580 (55.8)	1556 (49.4)	1024 (69.6)	< 0.001
Chronic hepatitis (except HBV, HCV)	1560 (33.8)	1179 (37.4)	381 (25.9)	< 0.001
Gall bladder diseases	1690 (36.6)	989 (31.4)	701 (47.6)	< 0.001
Abdominal pain	1387 (30.0)	921 (29.2)	466 (31.7)	0.098
KUB diseases	928 (20.1)	524 (16.6)	404 (27.4)	< 0.001
Pancreas diseases	271 (5.9)	133 (4.2)	138 (9.4)	< 0.001
Others*	1780 (38.5)	1085 (34.5)	695 (47.2)	< 0.001

Abbreviations: HBV, hepatitis B virus; HCV, hepatitis C virus; KUB, kidney–urinary–bladder

Others* includes hepatomegaly, splenomegaly, abdominal or pelvic mass, and ascites.

For peer review only - http://bmjopen.bmj.com/site/about/guidelines.xhtml

Table 3. Comparison of treatment received by the <3 and ≥3 groups following ultrasonography surveillance

Variable	<3 group, n (%)	≥3 group, n (%)	*p* value
PEI	200 (6.4)	244 (16.6)	< 0.001
Hepatectomy	343 (10.9)	221 (15.0)	< 0.001
RFA	71 (2.3)	101 (6.9)	< 0.001
Liver transplantation	2 (0.1)	6 (0.4)	0.015
TACE	584 (18.5)	453 (30.8)	< 0.001
Chemotherapy	1169 (37.1)	677 (46.0)	< 0.001
Radiotherapy	647 (20.5)	334 (22.7)	0.097
5 years survival probability	7.7%	14.4%	< 0.001

Abbreviations: HBV, hepatitis B virus; HCV, hepatitis C virus; CKD, chronic kidney disease; CVA, cerebral vascular accident; CCI, Charlson comorbidity index; DU, duodenal ulcer; GU, gastric ulcer; EV, esophageal varices; PEI, percutaneous ethanol injection; RFA, radiofrequency ablation; TACE, transcatheter arterial chemoembolization; UGI, upper gastrointestinal tract.

For peer review only

Table 4. Results of the multivariate Cox proportional hazard regression of predictors of death among patients with liver cancer

Variable	H.R.	95% C.I.	*p* value
Male	1.14	1.07-1.22	<0.001
Age (HCC diagnosis) per 5 years	1.02	1.01-1.03	0.002
Diabetes	1.13	1.04-1.22	0.004
Hypertension	0.83	0.78-0.89	<0.001
CVA	0.78	0.72-0.84	<0.001
CKD	0.84	0.74-0.92	<0.001
Viral hepatitis (HBV, HCV)	0.79	0.72-0.88	<0.001
CCI (per 1 score)	1.07	1.05-1.08	<0.001
US indications			
3 times or more US	0.80	0.75-0.85	<0.001
KUB diseases	0.92	0.86-0.995	0.037
Gall bladder diseases	0.90	0.85-0.96	0.001
Chronic liver diseases (except HBV, HCV)	0.90	0.81-0.999	0.047
Pancreas diseases	0.80	0.71-0.91	<0.001
Complication of liver cancer			
UGI bleeding (GU, DU)	0.92	0.86-0.98	0.009
EV bleeding	0.90	0.83-0.97	0.008
Treatments			
TACE	0.82	0.76-0.89	<0.001
Radiotherapy	0.81	0.75-0.87	<0.001
RFA	0.79	0.68-0.93	0.004
Chemotherapy	0.71	0.67-0.76	<0.001
hepatectomy	0.67	0.61-0.73	<0.001
PEI	0.64	0.57-0.70	<0.001
Background			
Employment	0.89	0.83-0.95	<0.001
Northern area in Taiwan	0.91	0.85-0.97	0.003

Abbreviations: HBV, hepatitis B virus; HCV, hepatitis C virus; CKD, chronic kidney disease; CVA, cerebral vascular accident; CCI, Charlson comorbidity index; DU, duodenal ulcer; GU, gastric ulcer; EV, esophageal varices; PEI, percutaneous ethanol injection; RFA, radiofrequency ablation; TACE, transcatheter arterial chemoembolization; UGI, upper gastrointestinal tract; US, ultrasonography surveillance

For peer review only

Table 5. Results of the multivariate logistic regression of the predictors for receiving ultrasonography surveillance three times or more during the 2 years preceding a liver cancer diagnosis

Variables	O.R.	95% C.I.	p value
Male	0.60	0.52-0.69	<0.001
Diabetes	1.56	1.31-1.86	<0.001
Hypertension	1.17	1.01-1.34	0.03
CKD	1.60	1.30-1.96	<0.001
US indications			
Viral hepatitis (HBV or HCV infection)	3.66	2.76-4.85	<0.001
Gall bladder diseases	1.82	1.59-2.09	<0.001
KUB diseases	1.80	1.53-2.11	<0.001
Pancreas diseases	1.79	1.37-2.34	<0.001
Chronic hepatitis except viral hepatitis	1.75	1.31-2.34	<0.001
Other*	1.47	1.29-1.69	<0.001
Abdominal pain	0.81	0.70-0.93	0.004
Central area in Taiwan	1.16	1.01-1.34	0.04

Abbreviations: HBV, hepatitis B virus; HCV, hepatitis C virus; CKD, chronic kidney disease; CVA, cerebral vascular accident; CCI, Charlson comorbidity index; KUB, kidney–urinary–bladder; US, ultrasonography surveillance

Other* includes hepatomegaly, splenomegaly, abdominal or pelvic mass, and ascites.

For peer review only

Figure Legends

Figure 1. Study flowchart.

Figure 2. Smoothing curve of the frequency of ultrasonography during the 2 years preceding liver cancer diagnosis against 5-year survival probability after adjustment. (x-axis: frequency of ultrasonography during the 2 years preceding liver cancer diagnosis; y-axis: probability of 5-year survival)

Figure 3. Survival curves of the <3 group and ≥3 group.

Figure 4. Receiver operating characteristic curves of the predictors for receiving ultrasonography three or more times during the 2 years preceding liver cancer diagnosis.

For peer review only

For peer review only - http://bmjopen.bmj.com/site/about/guidelines.xhtml

References

1. GLOBOCAN 2012: Estimated Cancer Incidence, Mortality and Prevalence Worldwide in 2012. Available at : http://globocan.iarc.fr/Pages/fact_sheets_cancer.aspx. Accessed July 6, 2016.

2. Ministry of Health and Welfare. Statistics of Causes of Death, Volume1. Available at : http://www.mohw.gov.tw/cht/DOS/Statistic.aspx?f_list_no=312&fod_list_no=2747 Accessed January 18, 2016.

3. Parikh S, Hyman D. Hepatocellular cancer: a guide for the internist. *Am J Med* 2007; 120: 194-202.

4. Gomaa AI, Khan SA, Toledano MB, et al. Hepatocellular carcinoma: epidemiology, risk factors and pathogenesis. *World J Gastroenterol* 2008; 14: 4300-4308.

5. Chen DS, Hsu NH, Sung JL, et al. A mass vaccination program in Taiwan against hepatitis B virus infection in infants of hepatitis B surface antigen-carrier mothers. *JAMA* 1987; 257: 2597-2603.

6. Liu CJ, Kao JH. Hepatitis B virus-related hepatocellular carcinoma: epidemiology and pathogenic role of viral factors. *J Chin Med Assoc* 2007; 70: 141-145.

7. Ni YH, Chang MH, Huang LM, et al. Hepatitis B virus infection in children and adolescents in a hyperendemic area: 15 years after mass hepatitis B vaccination. *Ann Intern Med* 2001; 135: 796-800.

8. Chang MH, Chen CJ, Lai MS, et al. Universal hepatitis B vaccination in Taiwan and the incidence of hepatocellular carcinoma in children. Taiwan Childhood Hepatoma Study Group. *N Engl J Med* 1997; 336: 1855-1859.

9. Wait S, Chen DS. Towards the eradication of hepatitis B in Taiwan. *Kaohsiung J Med Sci* 2012; 28: 1-9.

10. Chiang C-J, Lo W-C, Yang Y-W, et al. Incidence and survival of adult cancer patients in Taiwan, 2002-2012. J Formos Med Assoc. (2015), http://dx.doi.org/10.1016/j.jfma.2015.10.011

11. Lok AS, Sterling RK, Everhart JE, et al. Des-gamma-carboxy prothrombin and alpha-fetoprotein as biomarkers for the early detection of hepatocellular carcinoma. *Gastroenterology* 2010; 138: 493-502.

12. Singal A, Volk ML, Waljee A, et al. Meta-analysis: surveillance with ultrasound for early-stage hepatocellular carcinoma in patients with cirrhosis. *Aliment Pharmacol Ther* 2009; 30: 37-47.

13. Bruix J, Sherman M, American Association for the Study of Liver D.

For peer review only - http://bmjopen.bmj.com/site/about/guidelines.xhtml

Management of hepatocellular carcinoma: an update. *Hepatology* 2011; 53: 1020-1022.

14. EASL-EORTC clinical practice guidelines: management of hepatocellular carcinoma. *J Hepatol* 2012; 56: 908-943.

15. El-Serag HB, Davila JA. Surveillance for hepatocellular carcinoma: in whom and how? *Therap Adv Gastroenterol* 2011; 4: 5-10.

16. Kansagara D, Papak J, Pasha AS, et al. Screening for hepatocellular carcinoma in chronic liver disease: a systematic review. *Ann Intern Med* 2014; 161: 261-269.

17. National Health Insurance Research Database (NHIRD), Taiwan. http://nhird.nhri.org.tw/date_01.html Accessed May 22, 2016.

18. Chen YC YH, Wu JC, Haschler I, Chen TJ, Wetter T. Taiwan's National Health Insurance Research Database: administrative health care database as study object in bibliometrics. *Scientometrics* 2011; 86(2): 365-380.

19. Lin CC, Lai MS, Syu CY, et al. Accuracy of diabetes diagnosis in health insurance claims data in Taiwan. *J Formos Med Assoc* 2005; 104: 157-163.

20. Charlson ME, Pompei P, Ales KL, et al. A new method of classifying prognostic comorbidity in longitudinal studies: development and validation. *J Chronic Dis* 1987; 40: 373-383.

21. Deyo RA, Cherkin DC, Ciol MA. Adapting a clinical comorbidity index for use with ICD-9-CM administrative databases. *J Clin Epidemiol* 1992; 45: 613-619.

22. Klabunde CN, Potosky AL, Legler JM, et al. Development of a comorbidity index using physician claims data. *J Clin Epidemiol* 2000; 53: 1258-1267.

23. Wu CY, Hsu YC, Ho HJ, et al. Association between ultrasonography screening and mortality in patients with hepatocellular carcinoma: a nationwide cohort study. *Gut* 2016; 65: 693-701.

24. Zhang BH, Yang BH, Tang ZY. Randomized controlled trial of screening for hepatocellular carcinoma. *J Cancer Res Clin Oncol* 2004; 130: 417-422.

25. Thein HH, Campitelli MA, Yeung LT, et al. Improved Survival in Patients with Viral Hepatitis-Induced Hepatocellular Carcinoma Undergoing Recommended Abdominal Ultrasound Surveillance in Ontario: A Population-Based Retrospective Cohort Study. *PLoS One* 2015; 10: e0138907.

26. Santi V, Trevisani F, Gramenzi A et al. Semiannual surveillance is superior to annual surveillance for the detection of early hepatocellular carcinoma and patient survival. J Hepatol 2010; 53: 291-297.

27. Santi V, Buccione D, Di Micoli A et al. The changing scenario of hepatocellular carcinoma over the last two decades in Italy. J Hepatol 2012; 56: 397-405.

28. Giannini EG, Cucchetti A, Erroi V et al. Surveillance for early diagnosis of hepatocellular carcinoma: how best to do it? World J Gastroenterol 2013; 19:

For peer review only - http://bmjopen.bmj.com/site/about/guidelines.xhtml

8808-8821.

29. Del Poggio P, Olmi S, Ciccarese F et al. Factors that affect efficacy of ultrasound surveillance for early stage hepatocellular carcinoma in patients with cirrhosis. Clin Gastroenterol Hepatol 2014; 12: 1927-1933 e1922.

30. Cucchetti A, Trevisani F, Pecorelli A et al. Estimation of lead-time bias and its impact on the outcome of surveillance for the early diagnosis of hepatocellular carcinoma. J Hepatol 2014; 61: 333-341.

31. Wang JH, Chang KC, Kee KM, et al. Hepatocellular carcinoma surveillance at 4- vs. 12-month intervals for patients with chronic viral hepatitis: a randomized study in community. *Am J Gastroenterol* 2013; 108: 416-424.

32. Greten TF, Papendorf F, Bleck JS, et al. Survival rate in patients with hepatocellular carcinoma: a retrospective analysis of 389 patients. *Br J Cancer* 2005; 92: 1862-1868.

33. Lee JM, Yoon JH, Kim KW. Diagnosis of hepatocellular carcinoma: newer radiological tools. *Semin Oncol* 2012; 39: 399-409.

34. Adami HO, Chow WH, Nyren O, et al. Excess risk of primary liver cancer in patients with diabetes mellitus. *J Natl Cancer Inst* 1996; 88: 1472-1477.

35. La Vecchia C, Negri E, Decarli A, et al. Diabetes mellitus and the risk of primary liver cancer. *Int J Cancer* 1997; 73: 204-207.

36. Fujino Y, Mizoue T, Tokui N, et al. Prospective study of diabetes mellitus and liver cancer in Japan. *Diabetes Metab Res Rev* 2001; 17: 374-379.

37. El-Serag HB, Tran T, Everhart JE. Diabetes increases the risk of chronic liver disease and hepatocellular carcinoma. *Gastroenterology* 2004; 126: 460-468.

38. Yu MC, Tong MJ, Govindarajan S, Henderson BE. Nonviral risk factors for hepatocellular carcinoma in a low-risk population, the non-Asians of Los Angeles County, California. *J Natl Cancer Inst* 1991; 83: 1820-1826.

39. Lonardo A, Ballestri S, Targher G, Loria P. Diagnosis and management of cardiovascular risk in nonalcoholic fatty liver disease. Expert Rev Gastroenterol Hepatol 2015; 9: 629-650.

40. Lonardo A, Loria P. Potential for statins in the chemoprevention and management of hepatocellular carcinoma. J Gastroenterol Hepatol 2012; 27: 1654-1664.

41. Magee C. Kidney disease and death from cancer. *Am J Kidney Dis* 2014; 63: 7-9.

42. Izzedine H, Perazella MA. Onco-nephrology: an appraisal of the cancer and chronic kidney disease links. *Nephrol Dial Transplant* 2015; 30: 1979-1988.

43. Non-alcoholic Fatty Liver Disease Study G, Lonardo A, Bellentani S et al. Epidemiological modifiers of non-alcoholic fatty liver disease: Focus on high-risk groups. Dig Liver Dis 2015; 47: 997-1006.

For peer review only

For peer review only - http://bmjopen.bmj.com/site/about/guidelines.xhtml

44. Rinaldi L, Nascimbeni F, Giordano M et al. Clinical features and natural history of cryptogenic cirrhosis compared to hepatitis C virus-related cirrhosis. World J Gastroenterol 2017; 23: 1458-1468.

45. Piscaglia F, Svegliati-Baroni G, Barchetti A et al. Clinical patterns of hepatocellular carcinoma in nonalcoholic fatty liver disease: A multicenter prospective study. Hepatology 2016; 63: 827-838.

46. Chen CH, Dai CY, Hou NJ, et al. Etiology, severity and recurrence of acute pancreatitis in southern taiwan. *J Formos Med Assoc* 2006; 105: 550-555.

47. Bruix J, Sherman M, Practice Guidelines Committee AAftSoLD. Management of hepatocellular carcinoma. *Hepatology* 2005; 42: 1208-1236.

48. Makuuchi M, Kokudo N, Arii S, et al. Development of evidence-based clinical guidelines for the diagnosis and treatment of hepatocellular carcinoma in Japan. *Hepatol Res* 2008; 38: 37-51.

49. Llovet JM, Di Bisceglie AM, Bruix J, et al. Design and endpoints of clinical trials in hepatocellular carcinoma. *J Natl Cancer Inst* 2008; 100: 698-711.

50. Takayama T, Makuuchi M, Kojiro M, et al. Early hepatocellular carcinoma: pathology, imaging, and therapy. *Ann Surg Oncol* 2008; 15: 972-978.

51. Ye SL, Takayama T, Geschwind J, et al. Current approaches to the treatment of early hepatocellular carcinoma. *Oncologist* 2010; 15 Suppl 4: 34-41.

52. Kuo MJ, Chen HH, Chen CL, et al. Cost-effectiveness analysis of population-based screening of hepatocellular carcinoma: Comparing ultrasonography with two-stage screening. *World J Gastroenterol* 2016; 22: 3460-3470.

53. Bertakis KD, Azari R, Helms LJ, et al. Gender differences in the utilization of health care services. *J Fam Pract* 2000; 49: 147-152.

For peer review only - http://bmjopen.bmj.com/site/about/guidelines.xhtml

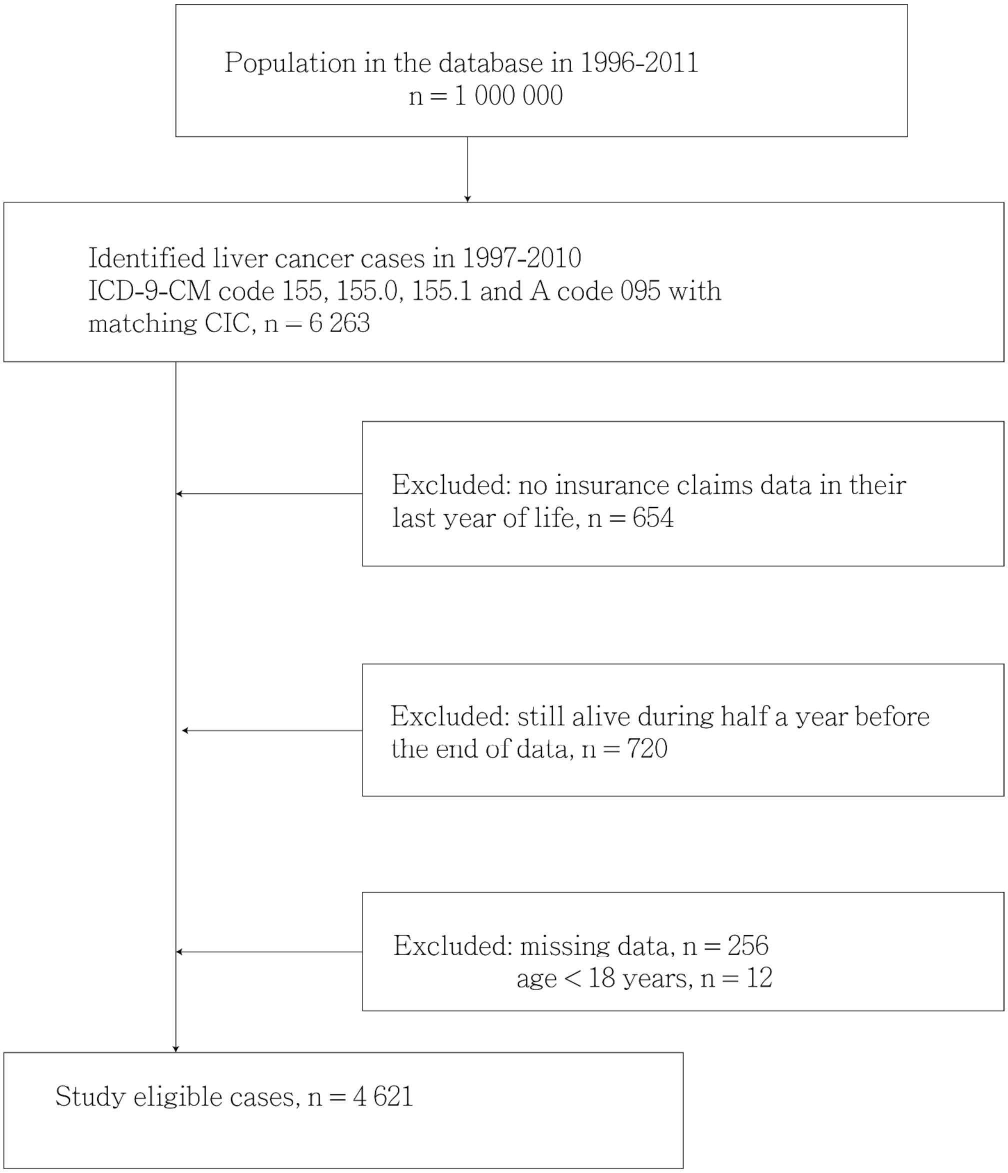

467x543mm (300 x 300 DPI)

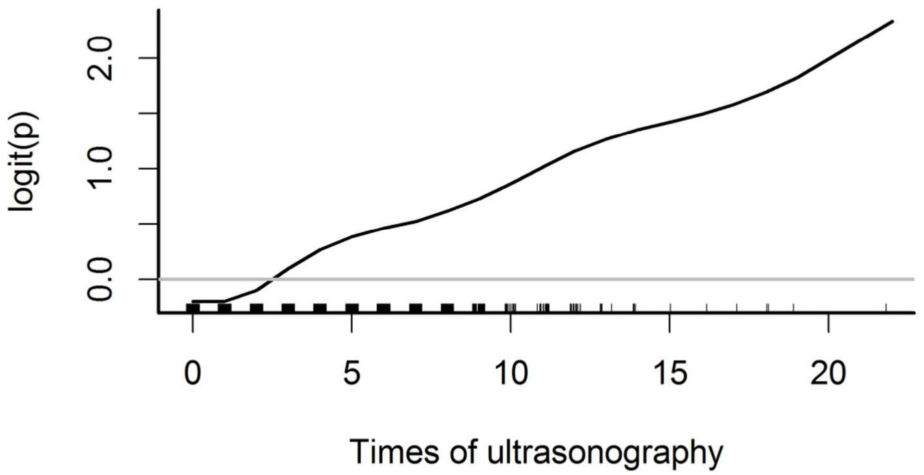

101x67mm (300 x 300 DPI)

view only

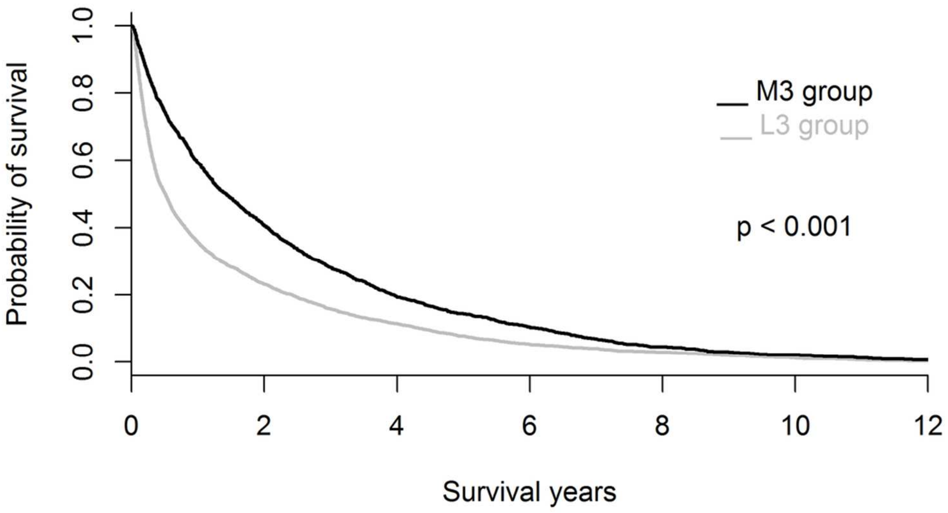

101x67mm (300 x 300 DPI)

view only

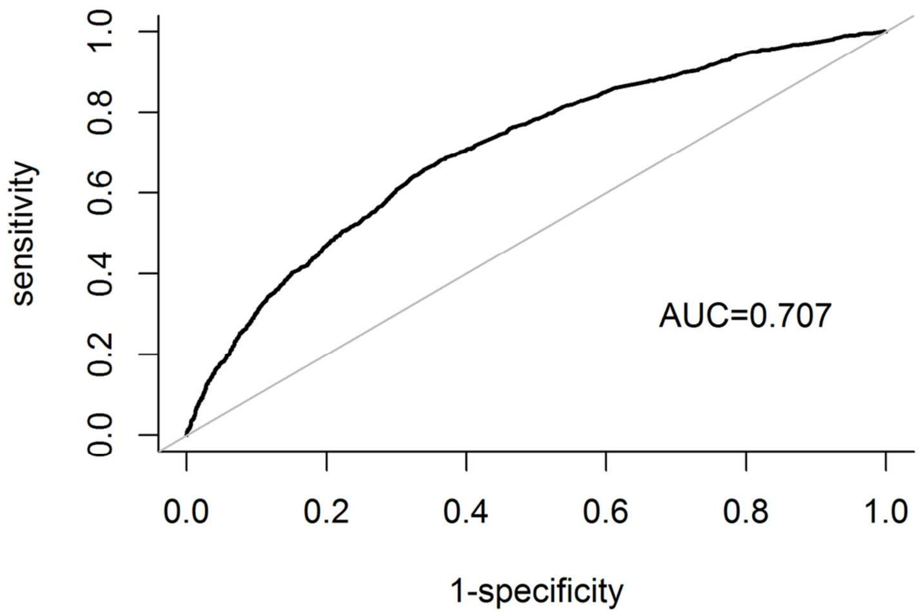

101x81mm (300 x 300 DPI)

For peer review only

STROBE Statement—Checklist of items that should be included in reports of ***cohort studies***

	Item No	Recommendation
**Title and abstract**	1	(*a*) Indicate the study's design with a commonly used term in the title or the abstract (page 2)
		(*b*) Provide in the abstract an informative and balanced summary of what was done and what was found (page 2)
**Introduction**		
Background/rationale	2	Explain the scientific background and rationale for the investigation being reported (page 5-6)
Objectives	3	State specific objectives, including any prespecified hypotheses (page 6)
**Methods**		
Study design	4	Present key elements of study design early in the paper (page 6)
Setting	5	Describe the setting, locations, and relevant dates, including periods of recruitment, exposure, follow-up, and data collection (page 6)
Participants	6	(*a*) Give the eligibility criteria, and the sources and methods of selection of participants. Describe methods of follow-up (page 7)
		(*b*) For matched studies, give matching criteria and number of exposed and unexposed (not indicated)
Variables	7	Clearly define all outcomes, exposures, predictors, potential confounders, and effect modifiers. Give diagnostic criteria, if applicable (page 7-8)
Data sources/ measurement	8*	For each variable of interest, give sources of data and details of methods of assessment (measurement). Describe comparability of assessment methods if there is more than one group (page 7-9)
Bias	9	Describe any efforts to address potential sources of bias (page 7)
Study size	10	Explain how the study size was arrived at (page 7)
Quantitative variables	11	Explain how quantitative variables were handled in the analyses. If applicable, describe which groupings were chosen and why (page 8)
Statistical methods	12	(*a*) Describe all statistical methods, including those used to control for confounding (page 9)
		(*b*) Describe any methods used to examine subgroups and interactions (page 9)
		(*c*) Explain how missing data were addressed (Page 7, figure 1)
		(*d*) If applicable, explain how loss to follow-up was addressed (page 7, figure 1)
		(*e*) Describe any sensitivity analyses (page 10)
**Results**		
Participants	13*	(a) Report numbers of individuals at each stage of study—eg numbers potentially eligible, examined for eligibility, confirmed eligible, included in the study, completing follow-up, and analysed (page 10)
		(b) Give reasons for non-participation at each stage (page 10, figure 1)
		(c) Consider use of a flow diagram (page 10, figure 1)
Descriptive data	14*	(a) Give characteristics of study participants (eg demographic, clinical, social) and information on exposures and potential confounders (page 11)
		(b) Indicate number of participants with missing data for each variable of interest (page 10, figure 1)
		(c) Summarise follow-up time (eg, average and total amount) (page 10)
Outcome data	15*	Report numbers of outcome events or summary measures over time (page 11)
Main results	16	(*a*) Give unadjusted estimates and, if applicable, confounder-adjusted estimates and their precision (eg, 95% confidence interval). Make clear which confounders were

For peer review only

		adjusted for and why they were included (page 11)
		(*b*) Report category boundaries when continuous variables were categorized (page 11)
		(*c*) If relevant, consider translating estimates of relative risk into absolute risk for a meaningful time period (page 13)
Other analyses	17	Report other analyses done—eg analyses of subgroups and interactions, and sensitivity analyses (page 13)
**Discussion**		
Key results	18	Summarise key results with reference to study objectives (page 13)
Limitations	19	Discuss limitations of the study, taking into account sources of potential bias or imprecision. Discuss both direction and magnitude of any potential bias (page 17)
Interpretation	20	Give a cautious overall interpretation of results considering objectives, limitations, multiplicity of analyses, results from similar studies, and other relevant evidence (page 13-14)
Generalisability	21	Discuss the generalisability (external validity) of the study results (page 14)
**Other information**		
Funding	22	Give the source of funding and the role of the funders for the present study and, if applicable, for the original study on which the present article is based (page 18)

*Give information separately for exposed and unexposed groups.

Note: An Explanation and Elaboration article discusses each checklist item and gives methodological background and published examples of transparent reporting. The STROBE checklist is best used in conjunction with this article (freely available on the Web sites of PLoS Medicine at http://www.plosmedicine.org/, Annals of Internal Medicine at http://www.annals.org/, and Epidemiology at http://www.epidem.com/). Information on the STROBE Initiative is available at http://www.strobe-statement.org.

For peer review only

For peer review only - http://bmjopen.bmj.com/site/about/guidelines.xhtml
